# The cyclin D1 carboxyl regulatory domain controls the division and differentiation of hematopoietic cells

**DOI:** 10.1186/s13062-016-0122-9

**Published:** 2016-04-29

**Authors:** Miguel Chaves-Ferreira, Gerald Krenn, Florence Vasseur, Aleksandr Barinov, Pedro Gonçalves, Orly Azogui, Ana Cumano, Zhi Li, Sandra Pellegrini, Benedita Rocha, Diego Laderach

**Affiliations:** INSERM, U 1020, U1151 – CNRS, UMR 8253, Institut Necker Enfants Malades, Faculté de Médecine Paris Descartes, 25, Rue du Dr Roux, Cedex 15 Paris, France; Institut Pasteur, 25, Rue du Dr Roux, Cedex 15 Paris, France; Present addresses: Instituto de Medicina Molecular, Avenida Prof. Egas Moniz, 1649-028 Lisbon, Portugal; IQUIBICEN-CONICET, Departamento de Química Biológica, Facultad de Ciencias Exactas y Naturales, Universidad de Buenos Aires, Intendente Guiraldes 2160, C1428EGA Buenos Aires, Argentina

**Keywords:** D cyclins, Hematopoiesis, Cell cycle

## Abstract

**Background:**

The family of D cyclins has a fundamental role in cell cycle progression, but its members (D1, D2, D3) are believed to have redundant functions. However, there is some evidence that contradicts the notion of mutual redundancy and therefore this concept is still a matter of debate.

**Results:**

Our data show that the cyclin D1 is indispensable for normal hematopoiesis. Indeed, in the absence of D1, either in genetic deficient mice, or after acute ablation by RNA interference, cyclins D2 and D3 are also not expressed preventing hematopoietic cell division and differentiation at its earliest stage. This role does not depend on the cyclin box, but on the carboxyl regulatory domain of D1 coded by exons 4–5, since hematopoietic differentiation is also blocked by the conditional ablation of this region.

**Conclusion:**

These results demonstrate that not all functions of individual D cyclins are redundant and highlight a master role of cyclin D1 in hematopoiesis.

**Electronic supplementary material:**

The online version of this article (doi:10.1186/s13062-016-0122-9) contains supplementary material, which is available to authorized users.

## Background

In mammalian cells, D type cyclins have a fundamental role in the initiation of the cell division in response to environmental signals. After exogenous stimulation, they are rapidly up regulated and form active holoenzyme complexes with their catalytic partners, the cyclin dependent kinases (CDK) 4/6. These complexes migrate to the nucleus in early-G1, where they monophosphorylate the retinoblastoma protein (Rb) [1]. Cyclins D/CDK4/6 activity is only required for transition through early G1 phase and across the restriction point into late G1 phase [1]. Progression through the late G1 phase and the G1/S transition are mediated by cyclin E/CDK2 complexes that phosphorylate Rb in other sites. Rb hyperphosphorylation disrupts its association with E2F transcription factor family members, allowing the transcription of several genes fundamental for cell cycle progression [Reviewed in [2]].

The cyclin D family comprises three members (D1, D2, D3), which have different tissue distribution. The role of each individual member of this family is yet subject of debate. Studies of mice deficient in individual cyclin D family members proposed that all D cyclins would have redundant functions. The role of each D cyclin in each tissue would therefore depend only of its expression in that tissue, a block of mouse development and hematopoiesis requiring the combined deficiency of all three D type cyclins [3].

Other evidence contradicts the notion of mutual redundancy. The D cyclins share a homologous cyclin box, but also have non-homologous domains. Numerous reports described that the cyclin D1 has major roles in the regulation of gene expression, not shared by other D cyclins. Such roles do not depend on the conserved cyclin box, but rather in the non-homologous carboxyl and amino terminal regions of D1, known as the D1 regulatory domains [4–7]. The D1 regulatory functions include the control of promoter accessibility [4, 6, 8, 9], the promotion of genome instability [7] and/or the contribution to DNA repair [10]. By genome-wide Chip analysis, D1 was found to bind up to 2840 putative sites of both proximal and distal promoters [7]. In particular, D1 bound the promoters of abundantly expressed genes, and controlled the expression of several important transcription factors involved in cell differentiation, including Notch1 [9] and NF-kB [11]. Of note, D1 binds to the promoters of cyclin D2 (*Ccnd2*) and cyclin D3 (*Ccnd3*) [9] suggesting that it could also be involved in their transcription, therefore having a major impact in initiating cell cycle progression. Results obtained in mice deficient in cyclin D3 further support that the functions of the individual members of the D cyclin family are not fully redundant. Since these mice express both D1 and D2, fully redundant functions of all D cyclins would be incompatible with the role of D3 in TN3-TN4 transition and in B cell differentiation [12, 13]. Besides, while the D1 deficient colony shows a very high mortality rate [14], colonies of D2 and D3 deficient mice do not [12, 15]. These observations suggest that each of the three D-cyclins may play unique, non-exchangeable cellular and tissular functions.

To address these controversies we studied mice previously described as D1 deficient, eliminated the expression of D1 by RNA*i*, overexpressed 4–5 in WT LSKs and generated conditional deficient mice which lack the D1 regulatory domains coded by exons 4–5. We here show that D1 regulates the expression of the mRNAs coding for D2 and D3, the absence of D1 preventing the division and differentiation of hematopoietic lineage cells. Overall, the data demonstrate a fundamental role of D1 in hematopoiesis, mediated by the carboxyl regulatory domain coded by exons 4–5.

## Results

### The hematopoietic differentiation in “D1 deficient” mice

The mice previously described as D1 deficient [14] (hereafter referred to as *Ccnd1*^Δ1-3^ mice) were produced by the insertion of a Neomycin cassette leading to the deletion of *Ccnd1* exons 1–3 (encoding the D1 N-terminal regulatory domain and the cyclin box) but sparing the *Ccnd1* promoter, the 5′ un-translated region, and the exons 4 and 5, these latter coding one of the D1 regulatory domains. This colony is known to have a high mortality rate [14]. To ensure *Ccnd1*^Δ1–3^ mice survival we had to develop unique breeding conditions (described in methods), since in standard breeding conditions we mainly obtained wild type (WT) or heterozygous mice. However, in spite of these conditions, in 684 mice born from crosses between heterozygous *Ccnd1*^1–3−/+^ mice the frequency of viable *Ccnd1*^Δ1–3^ mice (15 %) was lower than the 25 % predicted by Mendel laws, indicating prenatal mortality. A substantial fraction of homozygous mice also died in the first two-three weeks after birth.

**Fig. 1:**
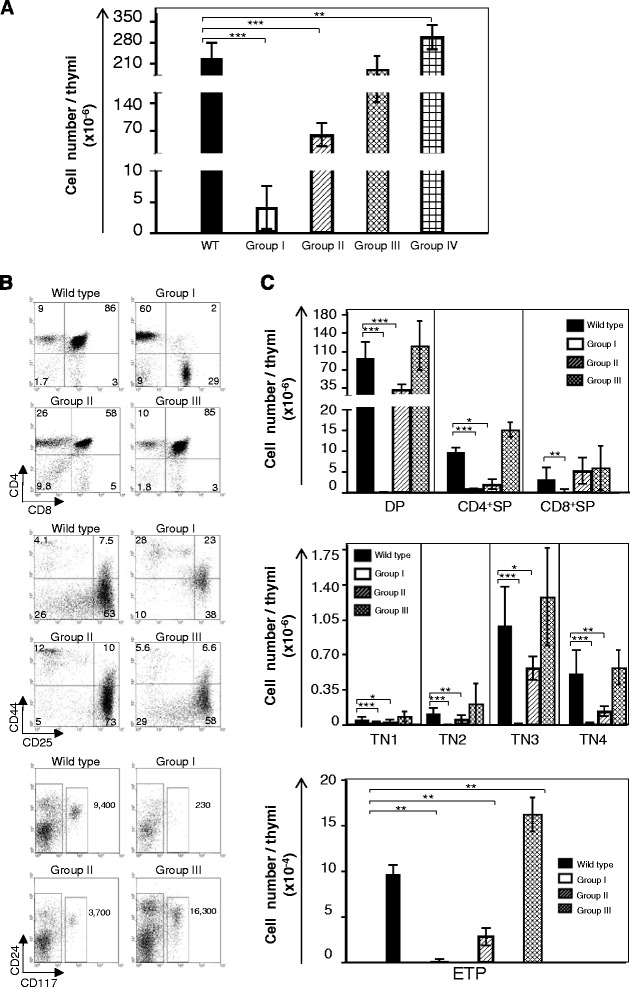
The thymocytes from 4 weeks old *Ccnd1* ^Δ1-3^ mice. **a** Thymocyte yields from different Groups of *Ccnd1* ^Δ1-3^ mice. The number of mice in each Group is shown in brackets: GrI (11), GrII (12), GrIII (25) and GrIV (10); results are expressed as the mean +/− SE. **b** The distribution of thymocyte populations. Dot plots are from a representative experiment in which 4-weeks-old littermates belonging to different Groups were studied simultaneously. Group IV is not shown, since 4-weeks-old mice do not have this Group. **c** Bar graphs represent the mean+/− SE from all mice we studied. The number of mice in each Group is shown in brackets: GrI (15) GrII (12) GrIII (26). Upper graphs: CD4/CD8 profiles in CD19^−^TER119^−^Gr-1^−^ thymocytes. Middle graphs: CD44/CD25 profiles in triple negative (TN: CD3^−^CD8^−^CD4^−^CD19^−^TER119^−^Gr-1^−^) cells. Lower graphs: CD44^+^ CD25^−^ TN1 populations, subdivided by their expression of CD24 and CD117. Numbers show the yields of ETPs (CD24^+^ CD117^+^)/thymus. Statistic significance: *p < 0.05; **p < 0.005; ***p < 0.001

Therefore, we first studied 4-weeks-old mice. In these mice, hematopoietic differentiation was very heterogeneous. Thymocyte numbers ranged from less than 0.5×10^6^ cells, to over 500x10^6^ cells. Based on the thymus size we subdivided this colony into four Groups (Fig. 1a). Group I mice had a severe thymus atrophy. The thymi had very few CD4^+^CD8^+^ (double positive-DP) cells (Fig. 1b, upper graphs). The distribution of triple-negative (TN: CD4^−^CD8^−^CD3^−^ Lineage^−^ thymocytes) subpopulations was abnormal. In contrast to WT mice, where the more mature CD44^−^CD25^+^ (TN3) and CD44^−^CD25^−^ (TN4) populations are prevalent [16], TN3 and TN4 thymocytes were virtually absent (Fig. 1b, middle graphs). Either the TN compartment was mostly constituted of CD44^+^CD25^−^ (TN1) and CD44^+^CD25^+^ (TN2) populations (Fig. 1b), or only had TN1 thymocytes (not shown). Since the TN1 thymocytes harbor different subpopulations, we further quantified the early thymocyte progenitors (ETP), believed to be the progenitors of all thymocyte sets and characterized by the co-expression of CD24 and c-kit (CD117) [16]. We found that in Group I mice ETPs were reduced up to 50 fold when compared to WT mice (Fig. 1b, lower graphs). Therefore, the *Ccnd1*^1-3^ deficiency may block thymus differentiation at its earliest stage. The major reduction of the ETPs further suggests that differentiation blocks may precede thymus seeding, *i.e*., may already be present in progenitors located in the bone marrow (BM).

Group II mice had moderate thymus atrophy. The thymocyte populations were enriched in TN cells and had fewer DP cells. The block on TN differentiation occurred at a later stage, *i.e*., in the TN3 to TN4 transition. Analysis of the TN1 population, however, showed a reduction of the ETP compartment, although less important than that found in Group I mice (Fig. 1b). Finally, in Group III (Fig. 1b) and Group IV (not shown) the thymocyte sub-populations distribution appeared similar to that of WT mice. However, the analysis of the TN1 compartment showed that these mice were also abnormal. In contrast to previous Groups where ETP populations were reduced, these mice had more ETPs than their WT littermates (Fig. 1b, lower graphs).

**Fig. 2:**
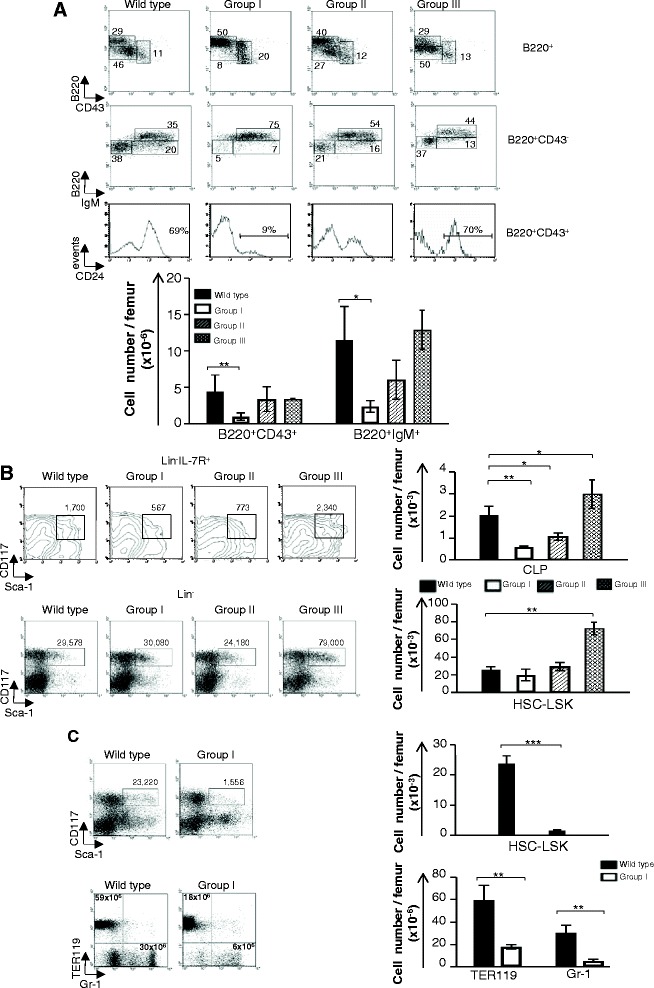
The bone marrow cells from *Ccnd1* ^Δ1-3^ mice. **a**, **b** Dot blots are from one representative experiment showing 4-weeks-old littermates belonging to different Groups studied simultaneously. Bar graphs represent the yield/femur of the different cell populations and are the mean+/− SE of all the mice we studied: GrI (15) GrII (12) GrIII (18). **a** B220^+^ B lineage cells. **b** Lineage negative (Lin^−^: CD3^−^CD4^−^CD8^−^CD19^−^TER119^−^Gr1^−^Mac-1^−^NK1.1^−^) progenitors analyzed according to their expression of IL-7R, Sca-1 and CD117. Upper graphs: CLPs; Lower graphs: LSK/HSCs. The numbers in the dot-plots represent yields/femur. **c** Dot-plots are from one representative experiment showing 9-days-old WT and *Ccnd1* ^Δ1-3^ littermates. Bar graphs are the mean+/SE of yields/femur all the 9-days-old mice we studied (*n* = 3). Upper graphs show LSK/HSC, lower graphs erythroid and myeloid lineage cells. Statistic significance: *p < 0.05; **p < 0.005; ***p < 0.001

B cell differentiation in the BM progresses from pre-pro B (B220^low^ CD43^+^ CD24^−^) to pro B (B220^low^ CD43^+^ CD24^+^) to pre-B (B220^low^ CD43^−^) to naïve B cells (B220^high^ IgM^+^) [17]. In *Ccnd1*^Δ1-3^ mice, B cell lineage differentiation blocks paralleled those found in the thymus (Fig. 2). Group I mice had a major pre-pro B differentiation block. Most B lineage cells were B220^low^ CD43^+^ CD24^−^ pre-proB precursors. Group II mice showed a partial pre-proB block and a reduction in pre-B cells, while in Groups III, IV B cell differentiation appeared normal (Fig. 2a). Earlier lymphoid lineage precursors were also affected. Group I mice had rare Lineage^−^IL-7R^+^Sca-1^+^CD117^+^ common lymphocyte progenitors (CLPs), and in the Group II mice the number of CLPs was also reduced, albeit to lesser extent. In contrast LSKs (Lineage^−^ Sca-1^+^CD117^+^) and myeloid and erythroid lineages were not affected. Surprisingly, further analysis of these precursors revealed that Group III was also abnormal. The number of CLP and LSK were actually increased as compared to WT littermates (Fig. 2b).

### The high mortality in the *Ccnd1*^Δ1-3^ colony associates with a deficiency of hematopoietic precursors and a high expression of D1 during the fetal and perinatal period

To identify the reasons justifying the high prenatal and perinatal mortality in this colony we studied 9-days-old mice. At this age the majority of *Ccnd1*^Δ1-3^ mice had a Group I phenotype. Moreover, besides the modifications described above in 4-weeks-old Group I mice, 9-days-old *Ccnd1*^Δ1-3^ mice also had an additional severe depletion of LSKs and erythroid and myeloid lineage cells (Fig. 2c). These results demonstrate that the *Ccnd1*^1-3^ deficiency already affects LSK generation. The deficiency in LSKs, erythroid and myeloid lineage cells is sufficient to explain the death of homozygous mice, since either lineage is required for survival. These results do not exclude additional roles of D1 in other organs, which may contribute the high mortality rate in this colony.

These results raise several questions: (i) Why has the *Ccnd1*^1-3^ deletion a higher effect during the embryonic and perinatal period?; (ii) How can a single deletion associate with three very different phenotypes?; (iii) How does the deletion of a single member of the cyclin D family affect hematopoietic differentiation, since other D cyclins, (that should guarantee cell division/differentiation), are still present?; (iii) How do some mice survive the effects of the *Ccnd1*^1–3^ deletion, i.e.*,* what are the “compensatory mechanisms” involved?

**Fig. 3:**
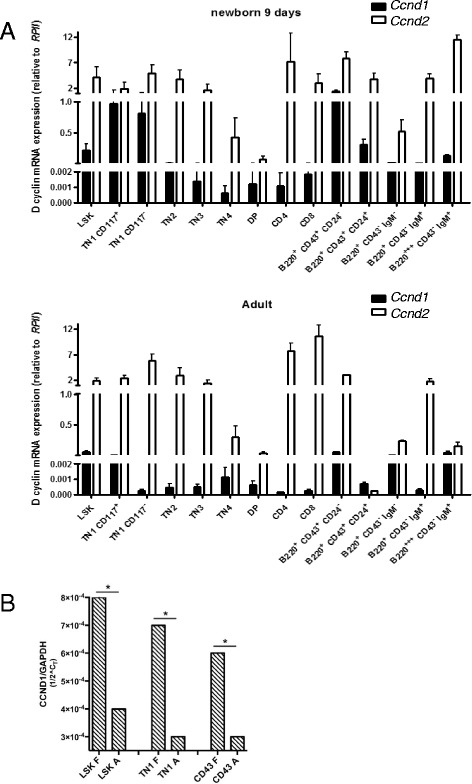
The expression of *Ccnd1* in mice of different ages. The individual subpopulations of hematopoietic lineage cells were sorted from WT mice with different ages (**a**). Quantification of *Ccnd1* and *Ccnd2* transcripts in different subpopulations of hematopoietic cells from 9-days and 4-weeks-old WT mice. Results show the mRNA expression of the *Ccnd1* and *Ccnd2*, determined by RT-qPCR, as referred to the *RPII* house-keeping gene. They are the mean+/− SD of three independent samples. Please note: Since the different PCR amplifications had the same efficiency, the expression levels of individual *Ccnd* can be directly compared. **b** Quantification of *Ccnd1* in LSK, TN1 and CD19^+^IgM^−^CD43^+^ precursors of 15-days-old fetus (F) and 4-weeks-old adult (A) mice, as referred to the *GAPDH* house-keeping gene. Results show the mean+/− SD of from three independent samples. SD deviations are too small to be visualized with the present scale. Statistic significance: *p < 0.05 (paired student *t*-test)

To address the first question we studied if the major effect of the *Ccnd1*^1-3^ deficiency during the pre-natal and perinatal periods was due to different expression levels of *Ccdn1*. We first quantified *Ccdn1* in comparison with the more abundant *Ccnd2* in hematopoietic cells obtained from 9-days-old and 4-weeks-old WT mice. As both PCR amplifications have the same efficiency, the expression levels of both cyclins can be directly compared. Interestingly, although *Ccnd1* was expressed in all hematopoietic lineage cells, there were major differences in the expression levels with age; *Ccnd1* was expressed at higher levels in immature precursors during the neonatal period (Fig. 3a). Its relative expression in hematopoietic progenitors declined with age. We next studied *Ccnd1* expression in different hematopoietic precursors (LSKs, TN1 and CD19^+^CD43^+^ B cell precursors) of 15-days-old WT embryos, the earliest time point when all these precursors can be identified. We found that *Ccnd1* was also highly expressed in these precursors, when compared to adult mice. The relatively high expressions of *Ccnd1* during the fetal and perinatal periods explain the major impact of the *Ccnd1*^1-3^ deficiency during these ages (Fig. 3b).

The age-related differences of the relative expression of D1, as well as the evolution of *Ccnd1*^Δ1-3^ mice phenotypes with age, explained how modifications in a single genetic allele give rise to mice with several phenotypes. In 9-days-old mice, hematopoietic multi-potent precursors express high levels of D1. Therefore, LSKs and all hematopoietic lineage cells are affected by this deletion, as found in 9-days-old Group I mice. In surviving mice, LSKs undergo a “compensatory” mechanism, since LSKs numbers are no longer reduced in 4-weeks-old Group I mice. Moreover, these “compensated” LSKs are competent as shown by their ability to generate both myeloid and erythroid lineage cells, present at normal levels in 4-weeks-old mice. Once competent LSKs are generated, these cells will differentiate progressively into all hematopoietic lineages, but this differentiation is known to take time. Therefore, the more immature cell sets will be generated first, explaining the phenotype of Group II mice, which reconstitute the immature B and T lineage cells, but are still deficient in more mature T and B lineage cells. Later on, the further differentiation of competent, “compensated” progenitors finally generates the more mature cell sets, as found in Group III mice. Thus, the same initial LSK deficiency that was “compensated” can generate the different *Ccnd1*^Δ1-3^ mice phenotypes. Each of these phenotypes actually represents “snap shots”, taken at different time points after the occurrence of the LSK “compensatory” event/s.

### The expression of D cyclins in *Ccnd1*^Δ1-3^ mice

To investigate the mechanisms responsible for the modifications in hematopoietic differentiation in *Ccnd1*^Δ1-3^ mice, as well as the compensatory mechanisms ensuring the survival of Group III mice, we studied how the various members of the D cyclin family were expressed in the different Groups of mice. Besides mRNA coding for cyclin D2 (*Ccnd2*) and D3 (*Ccnd3*) we also evaluated the expression of *Ccnd1* exons 4*–*5, since these domains are not deleted in *Ccnd1*^Δ1-3^ mice and were shown to have transcription regulatory activities.

Several mechanisms could explain the expression of a truncated D1 molecule, coded by these 2 exons. It is well known that the introduction of the neomycin cassette as a strategy to generate deficient mice induces perturbations at and around the insertion locus [18]. Current gene ablation strategies always delete the neomycin inserts, but this was not done in *Ccnd1*^Δ1-3^ mice. The neomycin cassette has potential transcription initiating sites, which could lead to the transcription *Ccnd1* exons 4–5. Moreover, *Ccnd1* exon 4 is known to have transcription-initiating sites allowing transcription of exons 4–5. In humans (in which D1 has 85 % homology with mouse D1) these two exons, as well as exon 5 alone can be transcribed independently [19].

**Fig. 4:**
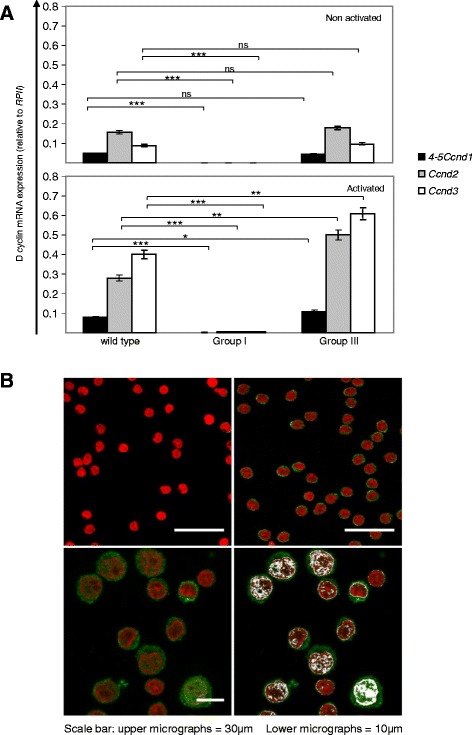
The expression of D cyclins in *Ccnd1* ^Δ1-3^ mice. **a** Expression levels of *Ccnd2*, *Ccnd3*, and exons 4–5 of *Ccnd1* relative to the *RPII* housekeeping gene. Results are from sorted naïve CD8^+^T cells before (*upper graphs*) or after 24 h stimulation with anti-CD3 mAbs (*lower graphs*). *RPII* housekeeping gene expression levels were similar in all mouse Groups. Results are the mean^+^/_−_ SD of three independent experiments. Statistic significance: *p < 0.05; **p < 0.005; ***p < 0.001 (paired student *t*-test). In each experiment amplifications from different Groups were performed simultaneously. **b** Expression of truncated 4–5 D1 protein by resting CD8^+^ T cells from Group I (*upper left panel*) and Group III mice (*upper right panel*); by activated CD8^+^ T cells from group III mice (lower panels): lower right panel show 4–5 migration into the nucleus and lower left panel the interaction with the DNA (in *grey*) upon T cell activation. The 4–5 D1 fragment was identified with rat anti-mouse D1 4–5 fragment mAb from ProMab Biotechnologies, Richmond, USA, revealed with a goat anti-rat Ab (Molecular Probes). The nucleus was labeled with DAPI and is shown in Red. Co-localization analysis was performed using the Image J program

We compared *Ccnd* expression in CD8^+^ T cells before and after activation with anti-CD3ε mAbs. We chose these cells to analyze a homogeneous population, preventing the variations in the relative expression of D1 in different cell types (Fig. 3). Moreover, CD8^+^ T cells facilitate the study of *Ccnd* expression after activation, since these cells can be readily activated in vitro with anti-CD3ε mAbs. In Group I mice, *4*–*5 Ccnd1*, *Ccnd2* and *Ccnd3* were virtually undetectable in both resting and activated cells (Fig. 4a). These results indicate that Group I mice are fully D1 deficient and, besides, that they also do not express other D type cyclins. By contrast, resting cells from Group III “compensated” mice transcribed 4–5 *Ccnd1, Ccnd2* and *Ccnd3* at WT levels. After activation, these mRNAs expression levels of were upregulated to higher levels than in WT cells (Fig. 4a).

Besides the quantification of mRNA expression, we also studied protein expression by confocal microscopy. For that purpose, we used a mAb raised by immunization of rats with a polypeptide from the C terminal mouse D1 molecule. This mAb fails to cross react with either with D2 or D3 [20]. To demonstrate the specificity of this mAb, we studied in parallel CD8 T cells from group I and group III mice. The CD8 T cells from Group I mouse failed to show any labeling, while all CD8 T cells from Group III mice were labeled (Fig. 4b, Upper graphs). These results demonstrate that this mAb only recognizes D1 4–5. Indeed, if this mAb would recognize other proteins besides D1, one would expect some labeling in cells from Group I mice, but no labeling was detected, demonstrating that this mAb only recognizes D1. By contrast, this mAb labels all cells of compensated mice. Since compensated mice are D1^1–3^ deficient, this mAb cannot label 1–3 that is not there, it must label 4–5. We conclude that compensated mice do express 4–5 proteins, which migrated to the nucleus after activation (Fig. 4b, lower left panel) and interacted directly with the DNA (Fig. 4b, lower right panel).

Therefore, while Group I mice are fully D1 deficient, Group III “compensated” mice express a truncated D1 molecule corresponding to the C-terminal regulatory domain, coded by the *Ccnd1* exons 5 or 4–5. Due to the rarity of *Ccnd1*^Δ1-3^ mice and the variability of their phenotype we could not recover sufficient protein to perform WB in these mice. We thus determined if the independent expression of 4–5 was a normal process also occurring in WT mice. Western blot analysis of CD8^+^ WT cells using an polyclonal Ab recognizing the C-terminal D1 region, shows the presence of two proteins with a MW of about 14 and 16.5Kd as expected from the independent translation of exons 5 and exon 4–5 respectively (Additional file 1: Figure S1). With this Ab, a longer exposure time was required to reveal the 34Kd 1–5 D1 protein (not shown). These results suggest that the independent expression of various isoforms of D1 may not be a peculiarity of *Ccnd1*^Δ1-3^ mice, or of human transformed cells [19]; it may be an event also occurring in WT cells.

**Fig. 5:**
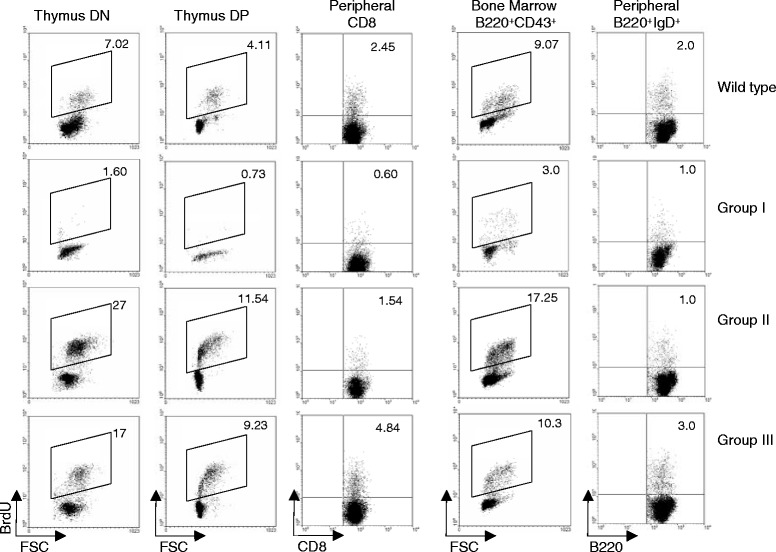
Division rates of hematopoietic lineage cells from *Ccnd1* ^Δ1-3^ mice. Results show BrdU incorporation in different cell types, defined at the top of each column. In thymocytes and bone marrow cells, BrdU incorporation was evaluated after a 2 h pulse. In peripheral T and B cells, BrdU incorporation was studied in mice receiving 2 BrdU injections/day spaced by twelve hours during three days. Barriers for positive cells were established in the same populations from mice that were not injected with BrdU, labeled simultaneously. Results are from one experiment out of 4 giving the same results. Please note: Since each mouse must be studied individually we could not evaluate BrdU incorporation in CLP or LSKs, which are too rare to allow identification of BrdU labeling in individual mice

These results provide a likely explanation for the different phenotypes of *Ccnd1*^Δ1-3^ mice. Group I mice are fully D1 deficient, but also do not express *Ccnd2* and *Ccnd3*. These results indicate that the D1 cyclin controls the transcription of *Ccnd2* and *Ccnd3* in hematopoietic cells, as suggested by the highly significant binding of D1 to the *Ccnd2* and *Ccnd3* promoters [9]. To further confirm the absence of D cyclins in these mice, we studied the capacity of cells from Group I mice to divide. In the absence of all D cyclins, hematopoietic cells should be unable to divide. Indeed, this was the case. Group I mice showed a profound block in BrdU incorporation in T cell precursors in the thymus and B cell precursors in the BM, mature T and B cells yet showing reduced division rates (Fig. 5). In contrast, the remaining Groups express truncated 4–5 D1 molecules. In response to environmental stimuli these cells show a major up-regulation of *4*–*5 Ccnd1* and other D cyclins, which could eventually lead to an increased division rate and lymphoid hyperplasia. Indeed, we found an increased BrdU incorporation in cells from Groups II and III mice (Fig. 5). By contrast we found no evidence of increased cell death in any of these populations (Additional file 2: Note 1 and Additional file 1: Figures S2 & S3).

These results indicate that the cyclin D1 is a master regulator of hematopoiesis. Moreover, the high division rates in mice expressing D1 4–5 suggest that this regulatory domain is responsible for controlling the expression of D2 and D3, and thus the hematopoietic division and differentiation. We thus used other strategies to demonstrate these hypotheses directly.

### Elimination of the full *Ccnd1* blocks hematopoietic differentiation

**Fig. 6:**
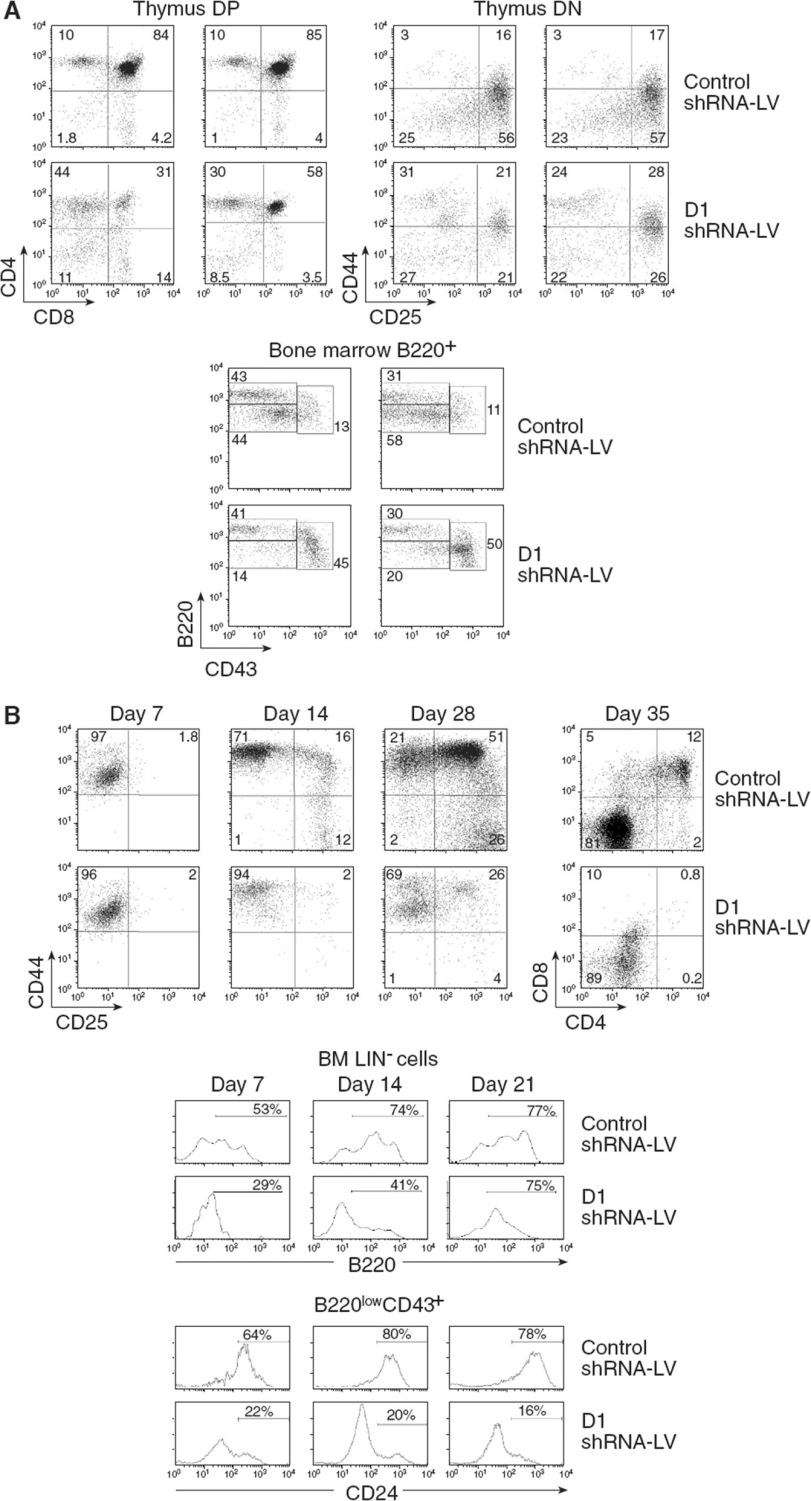
Effects of cyclin D1 RNA*i* in LSK differentiation. Bone marrow LSK (Lin^−^Sca1^+^ ckit^high^) progenitors were sorted from CD45.2+ mice BM, and were transduced with either a cyclin D1 or a control shRNA-LV-EGFP. **a** They were directly injected into lethally irradiated (1,700 rad) CD45.1^+^Rag^−^ mice. Results show EGFP^+^ cells in four different individual mice two months after LSK transfer. *Upper graphs*: EGFP^+^ thymocytes; *Lower graphs*: B cell lineage profiles in the BM, analyzed as described in Fig. 2. **b** They were cultured in in vitro conditions promoting T cell (*upper graphs*) or B/myeloid cell differentiation (*lower graphs*). Results show the phenotype of EGFP^+^ at different time points after culture

Since Group I mice are fully D1 deficient and showed a major block in hematopoietic differentiation, cyclin D1 could be fundamental for hematopoietic differentiation. To directly demonstrate this, we eliminated the full *Ccnd1* by RNA interference. We generated several lentiviral constructs expressing small hairpin RNAs (complementary to *Ccnd1* or scrambled control) and EGFP as reporter. Using a T cell lymphoblastic cell line, we selected one of these constructs, which induced a 90 % down-regulation of *Ccnd1* expression (Additional file 1: Figure S4). We compared the in vivo and in vitro behavior of adult LSKs transduced with this construct or a control one. Inhibition of *Ccnd1* expression by RNA*i* reproduced the differentiation blocks found in Group I, fully D1 deficient mice. In vivo, the thymus DP compartment was much reduced. The TN differentiation was blocked at the TN1-2 differentiation stages (Fig. 6a) as found in Group I mice (Fig. 1b). In the BM, we found the same pre-pro-B differentiation block, pro-B and pre-B cells virtually disappearing (Fig. 6a). In vitro, when LSKs were differentiated into T lineage cells in the presence of the OP-9 Delta-like 4 stromal cell line, we failed to generate TN2 cells or DP thymocytes (Fig. 6b). When LSKs were cultured in B cell lineage differentiation conditions with the OP-9 cell line, we found a major block in the generation of B220^high^ cells; the vast majority of B220^low^ cells did not express CD24, *i.e*.*,* the pre-pro-B differentiation block found in Group I mice was reproduced (Fig. 6b). These results formally demonstrate the master role of the D1 cyclin in hematopoietic differentiation.

### Overexpression of *Ccnd1* exons 4–5 in hematopoietic precursors increases cell division

To demonstrate directly the role of the truncated D1 4–5 molecule in hematopoiesis, one could express the 4–5 truncated molecule in the hematopoietic precursors of group I mice, and study if this overexpression rescue their phenotype. However, these experiments were impossible to perform and, importantly, could not give reliable results. We could not recover enough precursors to achieve efficient infection and reconstitution. Group I mice are quite young (what reduces the total number of cells we can recover from the BM), virtually devoid of progenitors, and these progenitors do not divide. Besides, these mice are very rare excluding that we could pool cells from several Group I mice to perform these studies. But more importantly, these experiments were unlikely to give straightforward conclusions. Since we had to wait several weeks before the analysis of the progeny of injected BM, and Group I rapidly compensate their phenotype, both transduced and non-transduced cells should show compensated phenotypes when tested (Additional file 2: Note 2). Therefore, to correlate 4–5 expression to hematopoietic cells division we enforced the expression of 4–5 in LSKs from WT mice. LSKs overexpressing 4–5 or infected with an empty vector were co-injected at the same number in lethally irradiated Rag^−^ mice. In the 4 mice we studied in two independent experiments, the proliferation rate of peripheral CD8^+^ T cells generated from 4–5 enforced progenitors was higher (*p* = 0.01) than that of mock transduced cells (Table 1). These results show that overexpression of 4–5 can modify division rates even in WT cells.

**Table 1:** *Ccnd1 4–5* over-expression increases lymphocyte division

BrdU+ CD8 + T cells (%)							
Experimental Group	Mouse #				Mean	SE	*p*
	1	2	3	4			
EGFP+ MOCK	20.0	13.5	10.7	30.6	18.7	6.6	0.01
EGFP+ D1 4-5	32.8	16.3	18.8	41.7	27.4	9.9	

LSKs were purified from 5FU-treated CD45.1 and CD45.2 wild type mice. CD45.1 LSK were transduced with a D1 4–5 coding retrovirus whereas CD45.2 LSK were transduced with the same control (Mock) virus. Equal numbers of each transduced LSK were injected simultaneously into lethally irradiated Rag^−^ mice. 3 weeks after cell transfer, the proliferation rates of the EGFP^+^ CD8^+^T subset were evaluated after 3 *i.p.* pulses of BrdU. Table 1 shows the % of BrdU^+^ CD8^+^T cells generated from transduced LSKs (EGFP^+^) Mock (CD45.2) and D1 4–5 (CD45.1) in each individual mouse (*n* = 4). Differences in proliferation between Mock and D1 4–5 transduced lymphocytes were evaluated by the paired Student *t*-test

### Conditional ablation of D1 4–5 prevents hematopoietic differentiation

The other possible alternative to evaluate the role of 4–5 in hematopoietic cell division and differentiation, was evaluating the effects of a deletion of 4–5. In these experiments it was fundamental to demonstrate that the deletion of 4–5 did not perturb the overall expression of the D1 molecule, *i.e*.*,* that by deleting 4–5 we were not just generating a fully D1 deficient mice. Therefore, we use an experimental strategy that should ensure the expression of D1 1–3 in 4–5 deleted mice. We generated conditional deficient mice by introducing two *loxP* sites flanking the 4^th^ exon (Additional file 1: Figure S5). In the selected strategy, after *Cre* recombination *exon 4* should be excised completely. The further splicing of exon 3 to the splice acceptor site of exon 5 introduces a 5′ stop codon, preventing the expression of the 5^th^ exon. This strategy was designed to allow the stable expression of exons 1–3, since neither the splice acceptor site nor the poly-adenylation sites of exon 5 are modified, which should guarantee mRNA stability.

We crossed these mice with mice expressing *Cre* under the *Vav1* promoter (which induces *Cre* expression in LSKs and all hematopoietic lineage cells) but had major problems in generating a viable progeny Vav-Cre *Ccnd1*^*4-5*^*flox*^*+/+*^ progeny (Additional file 2: Note 3). The very rare mice that had this genotype, expressed *Ccnd1 1–3* but not *Ccnd1 4*–*5* at early ages (4–7 days-old mice) (Additional file 1: Figure S6A). These results clearly demonstrated that the deletion of 4–5 did not perturb the overall expression of the D1 molecule, since exons 1–3 were yet transcribed. Western blot analysis in older mice using the DCS-6 mAb, that recognizes the D1 exon 3, confirmed the presence of a truncated molecule with a MW corresponding to the protein coded by *Ccnd1 exons 1–3.* It must be noted that we used this mAb previously, and showed that it is fully D1 specific, *i.e*., it does not recognize D2 or D3 or any other protein of the cell [20]*.* However, besides this truncated molecule, a band of 34Kd corresponding to the WT D1 protein was also present (Additional file 1: Figure S6B). To determine why the WT protein was still expressed in these mice, we studied the kinetics of *Cre* expression by RT-qPCR. While *Cre* expression remained stable in *Vav1-Cre* mice, it rapidly declined in *Vav1-Cre*^*+/−*^*Ccnd1*^*4-5*^*flox*^*+/+*^ mice (Additional file 1: Figure S6C). We conclude that these mice also compensated their *Ccnd1 exons 4–5* deficiency by the selection of the very rare hematopoietic cells failing to express *Cre*.

**Fig. 7:**
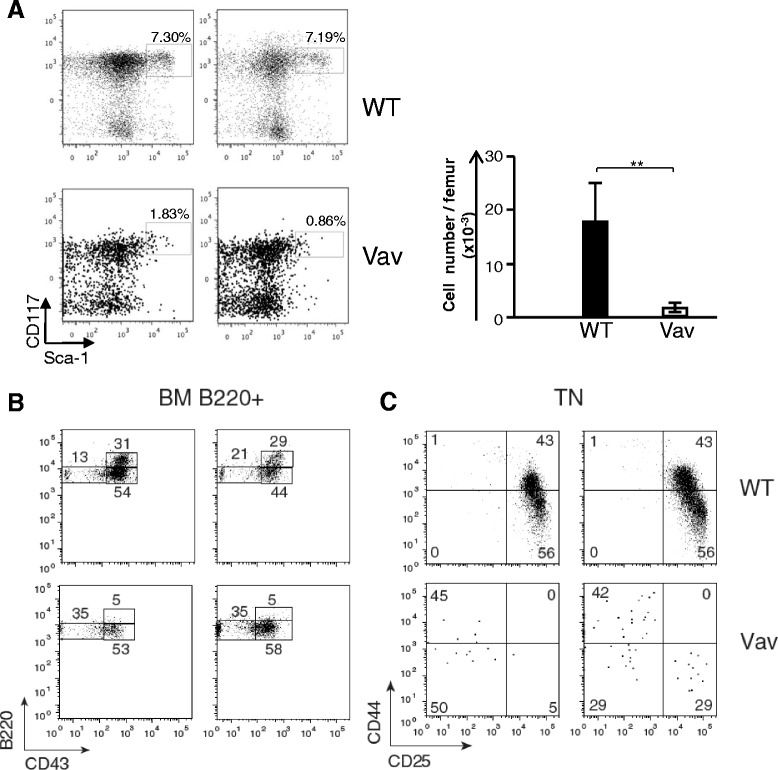
The progeny of WT and *Vav1-Cre* ^*+/−*^ *Ccnd1* ^*4-5*^ *flox* ^*+/+*^ LSKs in BM reconstituted chimeras. Lethally irradiated (1,700 rad) CD45.1^+^ CD45.2^+^ Rag2^−^ mice were injected *i.v.* with equal numbers (2,500) of LSKs from CD45.1^+^ WT and CD45.2^+^ *Ccnd1* ^*4-5*^ *flox* ^*+/+*^ mice, expressing the *Cre* recombinase under the *Vav1* promoter (Vav cells), and studied two weeks after BM transfer. *Upper graphs* show WT and *lower graphs* Vav cells (**a**). B220^+^ BM cells (**b**). TN thymocytes. Results are from two independent experiments

To study the role of *Ccnd1 4–5*, we thus performed competitive BM reconstitution experiments. To prevent the emergency of compensatory events, these chimeras were studied as early as at two weeks after BM transfer. When lethally irradiated CD45.1^+^/CD45.2^+^ Rag^−^ mice were injected with identical numbers (2,500 cells) of CD45.1^+^ WT and CD45.2^+^ LSKs from *Vav1-Cre*^*+/−*^*Ccnd1*^*4-5*^*flox*^*+/+*^ mice (hereafter referred as Vav cells), only 7 % of the hematopoietic lineage cells were of Vav origin. As expected, the frequency of CD45.2 Vav LSKs was much reduced when compared to that of CD45.1 WT LSKs (Fig. 7a). WT CD45.1 cells generated B220^high^ B lineage cells while Vav precursors only generated B220^low^ precursors enriched in B220^low^ CD43^−^ precursors (Fig. 7b), as found in Group I mice. In the thymus, all WT thymocytes showed a coordinated differentiation, TN precursors being at the CD44^+^CD25^+^ (TN2) and CD44^−^CD25^+^ (TN3) differentiation stages (Fig. 7c). Vav TN precursors in the thymus were virtually absent. Their CD44/CD25 phenotype did not reproduce that of WT TN cells (Fig. 7c-left) or they showed a major accumulation of the CD44^+^ CD25^−^ TN1 population, and a major reduction of TN2 and TN3 cells (right), as found in Group I mice (Fig. 1). These results confirm that the elimination of *Ccnd1 exons 4–5* blocks hematopoietic differentiation, reproducing the phenotypes of fully D1 deficient, Group I mice.

## Discussion

The present data emphasize the difficulty of evaluating the role of fundamental molecules since their ablation frequently increases mortality or/and leads to the emergency of compensatory mechanisms, which may mask ongoing major effects. Thus, the different phenotypes of *Ccnd1*^Δ1-3^ mice were initially very perplexing. It was not evident why the partial deletion of a single gene would generate mice with such different phenotypes as Group I, II, III mice. The reasons behind this heterogeneity became clear, once we associated the mortality rates in this colony at different ages, with the variations in both phenotypes and D1 expression levels. A substantial fraction of *Ccnd1*^Δ1-3^ embryos died, indicating that this deficiency can be embryonic lethal. Another large fraction of mice died shortly after birth. The comparison of 9-days and 4-weeks-old mice identified that the *Ccnd1*^1-3^ deletion affected LSKs in 9-days-old mice, inducing severe reductions in erythroid and myeloid lineages. Thus, mice often died during this period, since either erythroid or myeloid lineages are required for survival. The higher pre-natal and perinatal death of these mice is also justified by a much higher expression of *Ccnd1* during these periods, when compared to adult mice. After this critical pre- and peri-natal period, mice survived if they reconstituted LSKs, erythroid and myeloid lineages. These results indicated firstly that the *Ccnd1*^1-3^ deletion already affected LSK generation, and secondly, that *Ccnd1*^Δ1-3^ mice developed one or several “compensatory” mechanisms overcoming the LSK deficiency. This compensation not only increased LSK numbers, but also generated competent LSKs as shown by their capacity to reconstitute the erythroid and myeloid lineages in 4-weeks-old mice. Moreover, “compensation” did not occur simultaneously in all mice.

The asynchronous generation of competent LSKs in *Ccnd1*^Δ1-3^ mice was sufficient to fully explain the different phenotypes of individual mice from this colony. Once competent LSKs are generated, these cells differentiate progressively though this differentiation takes time. Therefore, they will first generate the more immature hematopoietic cell sets. This explains the phenotype of the Group II mice, which reconstitute the most immature, but are still deficient in more mature, T and B lineage cells. Later, the further differentiation of these competent progenitors will lead to the generation of more mature cells, as found in “fully compensated” Group III mice, whose phenotype is largely equivalent to that of normal mice. Thus, the same initial LSK deficiency, when “compensated” at different time points in different mice, is expected to generate the three different phenotypes, which correspond to “snap-shots” taken at different time points after the occurrence of the LSK “compensation”.

Our results also show why D1 is fundamental for hematopoietic differentiation, and how “compensation” occurs. We demonstrated that the hematopoietic differentiation deficiency in Group I mice was indeed due to the D1 deficiency, since we reproduced the same hematopoietic differentiation blockages by eliminating the full D1 molecule by *RNAi*. Concerning the mechanisms, we found that fully D1 deficient Group I mice did not transcribe *Ccnd2* and *Ccnd3*. The absence of all members of the D cyclins family in these mice is also confirmed by a failure of their cells to divide, *i.e*., to incorporate BrdU. Indeed, all subpopulations of hematopoietic lineage cells from Group I mice showed a virtual absence of BrdU incorporation, while we found no evidence that increased apoptosis contributed to their reduced numbers, or to the hematopoietic differentiation blockage. These results indicate that D1 controls the expression of D2 and D3, by regulating their transcription.

The role of D1 in the transcription of *Ccnd2* and *Ccnd3* was not unexpected. It is well known that D1 has major roles in the regulation of gene transcription. These roles do not depend on the conserved cyclin box domain, but rather on the 3′ and 5′ regulatory domains. In genome-wide Chip analysis, D1 was found to bind up to 2840 putative sites of both proximal and distal promoters [7]. In particular, it was shown that D1 binds the *Notch 1* promoter and regulates the expression of Notch 1 [9]. Since D1 binds the *Ccnd2* and *Ccnd3* promoters with a twenty fold higher significance (*p* value) than the *Notch 1* promoter [9], a role of D1 in transcribing *Ccnd2* and *Ccnd3* is to be expected.

Our analysis of compensated mice and of mice in which the expression of exons *4*–*5* was eliminated by homologous recombination indicates that the C-terminal region of D1, coded by *Ccnd1 exons 4–5*, regulates D2 and D3 transcription. This regulatory domain is well known to control gene expression [4–7]. This truncated D1 was not eliminated in the construction of *Ccnd1*^Δ1-3^ mice. Western blot analysis suggests that mature T cells from WT mice also express truncated D1 molecules coded by *Ccnd1 exons 5 and 4–5*, as it was reported in human cells [19]. Interestingly, these truncated molecules migrate to the nucleus after activation. Lastly, over-expression of these two exons in WT cells increased division rates, while the elimination of these exons by homologous recombination compromised mice survival, and LSKs from *Vav1-Cre*^*+/−*^*Ccnd1*^*4-5*^*flox*^*+/+*^ fared very poorly in BM reconstitution chimeras. Although these chimeras were injected with the same number of *Vav1-Cre*^*+/−*^*Ccnd1*^*4-5*^*flox*^*+/+*^ and WT LSKs, the number of *Vav1-Cre*^*+/−*^*Ccnd1*^*4-5*^*flox*^*+/+*^ LSKs was much lower than WT LSKs. Similar to full D1 deficient mice, *Vav1-Cre*^*+/−*^*Ccnd1*^*4-5*^*flox*^*+/+*^ LSKs showed a B-pro-B differentiation block and were unable to generate TN thymocytes, in contrast to WT LSKs.

## Conclusions

To summarize, our data shows that the D1 cyclin has a fundamental role in hematopoietic differentiation, being required for the expression of the other members of the D cyclin family and the generation of competent LSKs. Since D2 is the most abundant cyclin in hematopoietic lineage cells [21], the role of D1 cyclin as a master regulator of early hematopoiesis excludes the notion that individual D cyclin family members have fully redundant functions. Previous observations performed on *Ccnd3*^−/−^ mice support the same notion. Since these mice express both D1 and D2, fully redundant functions of all D cyclins would be incompatible with the role of D3 in TN3-TN4 transition and in B cell differentiation [12, 13]. However, the relative abundance of each D cyclin impacts on the effect of D type cyclin deficiencies. In both D1 and D3 deficiencies, the major impact in hematopoiesis correlated to a higher expression of these cyclins. Our results indicate a temporal and differential role of each D-type cyclin in hematopoietic differentiation. While D1 plays a role in earlier multi-lineage progenitors, D3 affects B cell differentiation and the TN3-TN4 transition in the thymus. The strong progenitor default induced by the *Ccnd1* deficiency causes that the vast majority of the deficient animals to die before birth or shortly after birth approaching the lethal phenotype of *Ccnd1*^Δ1-3^*Ccnd2*^−/−^*Ccnd3*^−/−^ mice [3].

## Methods

### Mice

CD45.2^+^C57BL/6 and CD45.1^+^/45.2^+^ Rag-2 deficient C57BL/6 mice were bred at the Center for Development of Advanced Experimentation Techniques, Orleans, France. Mice lacking the *Ccnd1 exons 1–3* (*Ccnd1*^Δ1–3^ mice) were kindly provided by Dr. Sicinski [14]. Mice expressing the *Cre* recombinase under the *Vav1* promoter were a kind gift of Dr. A. Potocnik [22]. This study was performed in strict accordance with the recommendations in the Guide for the Care and Use of Laboratory Animals of the Ministère de l’Agriculture, de la Pèche et de l’Alimentation, France. All of the animals were handled according to approved institutional animal care and use committee (IACUC) protocols of the Institut Necker Enfants Malades, Faculté de Médecine Paris Descartes, Paris, France. The Committee on the Ethics of Animal Experiments of the University Paris V approved the protocol.

### The breeding of *Ccnd1*^Δ1-3^ mice

Since *Ccnd1*^Δ1–3^ females are incapable of nursing due to an impaired mammary gland development, and the *Ccnd1*^Δ1–3^ males have low sperm counts [14], *Ccnd1*^Δ1–3^ mice were obtained by crossing heterozygous mice, in very special conditions we adapted for the D1 deficiency. It must be noted that these breeding conditions were fundamental to obtain Groups I and II mice. In standard breeding conditions most of the surviving progeny were WT or heterozygous, homozygous mice being of smaller size, died shortly after birth, the few survivals having compensated (Group III) phenotype. The major problems of this breeding and the strong selection pressure for “compensated” mice explain why Groups I and II mice were rare in previous colonies of *Ccnd1*^Δ1-3^ mice, being considered as outliers and not investigated further [14].

Briefly, the breeding was done exclusively in isolators and under continuous direct supervision. Since mothers frequently kill fragile pups and/or WT littermates outcompete them for feeding, *Ccnd1*^Δ1-3^ pups were identified by their neurological abnormalities during the first days after birth and transferred to WT foster mothers. We found that their neurological abnormalities prevented *Ccnd1*^Δ1-3^ mice from climbing to reach food and water, while the dental abnormalities prevented feeding and the ingestion of solid food. These handicaps may explain the reduced size reported in these mice. Thus, when pups reached three weeks of age the teeth were cut regularly and wet soft food was directly provided every day to the litter. Finally, couples were maintained for two litters only, since both fertility and the number of Group I mice declined with generations. Moreover, the maintenance of this colony in isolators was mandatory, since contra-selection of Group I mice occurred even with minor infections, which are not considered to lead to the loss of the s.p.f status. In contrast to previous reports [14] when bred in these conditions, the size and body weight of *Ccnd1*^Δ1-3^ mice was comparable to that of WT littermates.

### Antibodies for flow cytometry, western-blots and confocal analysis

The mAbs used for flow cytometry and/or cell sorting (BD Pharmingen) were anti: CD4, CD3, CD8, CD44, CD25, CD24, CD117, B220, CD43, Ter119, GR1, Sca-1, IL-7R, CD45.1, CD45.2, activated-Caspase-3 and Bcl-2. They were either directly conjugated to FITC, PE, APC, PerCP or biotin, these later being revealed by streptavidin-APC or PerCP5.5. To study cells in S phase in central lymphoid organs mice received two *i.p* injections of 1 mg of BrdU (Sigma) at a 4 h interval. To analyze proliferation in peripheral organs, mice were injected with 1 mg of BrdU *i.p.* every 12 h during 3 days. BrdU incorporation was detected using a FITC-conjugated anti-BrdU antibody (BD Pharmingen).

The Abs used for Western blots recognizing mouse Cyclin D1 used were: for the truncated 1–3 molecule, the mAb DCS-6 (CellSignal #2926) generated by immunization with a protein corresponding to aa 151–170, localized in the Exon 3. We had previously used this mAb extensively and confirmed it only recognized D1, and did not cross-react with D3 and D3 [20]; a polyclonal rabbit Ab recognizing the C-terminal part of the molecule (AbCam 7958).

To visualize the truncated D1 4–5 protein by confocal microscopy, CD8^+^ T cells were placed in slides previously coated with 0.1 % poly-L-Lysine (Sigma), then fixed with 2 % PFA, permeabialized with 0.15 % Triton and incubated with Image-IT Fx signal enhancer (Molecular Probes). These cells were then labeled with a rat anti-mouse mAb purchased at ProMab Biotechnologies, Richmond, USA. This mAb raised by the immunization of rats with a recombinant protein corresponding to the C terminal of mouse D1, not shared by D2 or D3 and the manufacturer provided us evidence it did not recognize D2 and D3. They were washed and incubated with a labeled goat anti-rat Ab (Molecular Probes). Slides were overlaid with coverslips in Vectashield® mounting medium (Vector) which contains DAPI for nuclear staining, shown in Red. Confocal microscopy was performed with the Zeiss LSM700 axioplan microscope (Carl Zeiss Micro-Imaging GmbH, Germany). The same cell populations incubated with the goat anti-rat Ab were used to determine background staining. The interactions between 4–5 polypeptide and the DNA were evaluated by the Image J program and are shown in grey.

### Western blot analysis

Western blots were performed with 3x10^6^ CD8^+^T cells that were lysed in modified RIPA buffer (50 mM Tris/HCl pH 8, 200 mM NaCl, 1 % Nonidet P40, 0.5 % deoxycholate, 0.05 % SDS, 2 mM EDTA), 1 mM orthovanadate and a protease inhibitor cocktail (Roche Diagnostics). A total of 30 μg of proteins were separated by 15 % SDS-PAGE. After blocking, membranes were incubated with either the anti-Cyclin D1 DCS-6 mAb recognizing both mouse and human D1 exon 3 or with an Ab against the C-terminal part of the molecule (AbCam 7958). Abs were diluted in 5 % non-fat milk, 1x PBS, 0,1 % Tween-20 and membranes incubated overnight at 4 °C with gentle shaking. Immunoblots were analyzed by ECL with the Western Lighting Chemiluminescence Reagent Plus (PerkinElmer) and revealed by Fuji Image Quant LAS-4000. For re-bloting with actin, blots were stripped in 0.2 M glycine (pH2.5) for 30 min at room temperature and re-bloted with monoclonal anti-actin (Sigma-Aldrich, clone AC-40). Intensity of the bands was analyzed using ImageJ 1.48v software.

### RT-qPCR and shRNA-Lentivirus

Primers used for RT-qPCR were selected in order to include at least one intron in the amplicon, no match with other murine genes by standard blast, no genomic amplification and for identical amplification efficiency. Results shown in Fig. 3b were obtained from samples of 50 sorted cells, using the single cell RT-qPCR techniques we described previously [23]. Oligonucleotide sequences are shown in Additional file 3: Materials.

For the production of lentivirus vectors we used the pLV-HTM plasmid (kindly provided by Dr. Trono Didier, Geneva University) which is a self-inactivation third generation HIV1-derived vector [24]. The annealed oligonucleotides coding for shRNA were ligated into *ClaI* and *MluI* double-restricted plasmid by standard cloning procedures, using restriction enzymes and T4 DNA ligase (New England BioLabs Inc). In these conditions, the production of siRNA is under the control of *H1* (RNA polymerase type III) promoter, and the reporter gene, the green fluorescent protein (*EGFP*) is expressed under the control of eukaryotic *EF-1α* promoter. All plasmids were verified by sequence analysis. Lentivirus particles were produced by transient transfection of 293 T cells according standard protocols. Briefly, subconfluent 293 T cells were co-transfected with 20 μg of plasmid vector, 15 μg of pCMVR8.74 and 5 μg of pMD.G envelope (VSVG) by calcium phosphate method. After overnight incubation, medium was changed for a fresh one and supernatants were harvested at 48 and 72 h. Supernatants were further concentrated (100X) by ultracentrifugation, titrated and stored at −80 °C until use. Viral titers expressed as TU/ml were determined by assessing transduction of 293 T cells with serial dilutions of virion preparations. Batches with titers ≥10^8^ TU/ml were used.

### Cloning of *Ccnd1* exons 4–5 and retrovirus production

RNA was extracted from purified lymph node CD8^+^T cells after 24-h anti-CD3 activation using RNeasy Plus kit (Qiagen) and retrotranscribed with MuLV from the GeneAmp RNA PCR kit (Applied Biosystems). This cDNA was used as template for the cloning PCR using the high fidelity proof reading Pfu Turbo Polymerase (Stratagen) with the cloning primers (see Additional file 3: Materials). The purified PCR product was subcloned in a passenger pCR®-Blunt II TOPO® vector (ThermoFisher Scientific) and sequenced. This passenger vector was further restricted with *MfeI* and *SnaBI* enzymes (New England Biolabs) and cloned into pMiev retrovirus vector. PlatE virus packaging cells were transfected with pMiev (empty or *Ccnd1* 4–5) in presence of lipofectamin LTX (Invitrogen). Media was changed at 24 h and supernatant harvested two days after, filtered onto 0.45 μ and used to transduce purified LSK.

### Purification, transduction and differentiation of hematopoietic progenitor cells

CD45.2^+^ (or alternatively CD45.1^+^) C57BL/6 mice were injected *i.v.* with 100 mg/kg 5FU (TEVA Pharma). Bone-marrow (BM) cells were recovered 5 days later and depleted of Lineage positive cells with a cocktail of rat mAbs (anti CD11b, CD8, CD5, CD45R/B220, Gr-1, Ter119) and anti-rat IgG conjugated dynabeads (Dynal). Hematopoietic progenitors were further enriched using the Spin Sep Purification kit (Stem Cell Technologies) following manufacturer instructions. Using this double step procedure, LSK progenitors were enriched 200 times with a recovery of 80 % of the initial progenitor cells after the procedure.

Transduction of enriched progenitors was performed in the presence of 5 μg/ml of protamine sulphate (Sigma) and 10 μg/cm^2^ coated RetroNectin® (Takara). Briefly, 1x10^6^ enriched progenitors were plated in 96 well plate in 200 μl of complete RPMI media (Gibco) in the presence of 10 ng/ml SCF, 50 ng/ml TPO, 100U/ml IL-6, 10 ng/ml IL-11 and 5 ng/ml Flt3-L, all recombinant murine cytokines from R&D Systems. Virus supernatants were added at a multiplicity of infection MOI = 10. Plates were centrifuged at 1000 g during 1 h at 20 °C and further cultured at 37 °C for other 4 h. At the end of this 5 h transduction protocol, cells were extensively washed and injected into lethally irradiated CD45.1^+^Rag^−^ mice or cultured “in vitro”, in conditions promoting either myeloid/B cells or T cells development. Myeloid and B cells development was performed by co-culture of these progenitors on OP9 stroma cell line whereas T cell development was induced by co-culturing progenitors on OP9-DL4 stroma (a kind gift of A. de la Coste) as previously described [25]. Briefly, OP9 or OP9-DL4 cells were seeded into 6-well tissue culture plates the day before the co-culture with progenitors. All cultures were performed in presence of 1 ng/ml IL-7 and 5 ng/ml Flt3-L (both recombinant murine cytokines from R&D Systems) and fed every 4 days. At the indicated time points, cells were harvested, counted and their phenotype evaluated by cytometry.

### Conditional deletion of *Ccnd1* exons 4–5

The *Ccnd1* exons 4-5^flox+/−^ mice line was established at the Mouse Clinical Institute (Illkrich, France) by transforming C57BL/6NTac embryonic stem cells with a linearized conditional targeting vector. This vector (shown in Additional file 1: Figure S5) was constructed to delete the *Ccnd1 exon 4*, by flanking it with two *loxP* sites, one introduced in intron 3 and another in intron 4. Deletion of the flanked exon by *Cre* mediated homologous recombination results in a frame-shift mutation of exon 5 with an early stop codon. This strategy was selected to maintain a normal expression of the *Ccnd1 exons 1–3*, since for the stability of the respective mRNA after splicing it was important to keep the splice acceptor site of exon 5 and the poly-adenylation signals, otherwise RNA decay was likely to occur. The neomycin resistance gene (*Neo*^*r*^) under the control of the *PGK* promoter (*PGK-Neo*^*r*^) was inserted into intron 4 upstream the *loxP* site. Two *FRT* sites flanked *pGK-Neo*^*r*^. The 3480 bp genomic fragment 5′ upstream of exon 4 and the 3509 bp 3′ of exon 4 were amplified by PCR and used as 5′ and 3′ homology arms. 186 neomycin resistant clones were screened for homologous recombination by PCR using 3 primer pairs, composed of external primers and vector specific primers. 6 positive clones were obtained. Positive clones were further confirmed by southern blot using a Neo^r^ probe and a 5′ external probe. Correctly targeted clones were transiently transfected with a Flp expression vector to delete the *PGK-Neo*^*r*^ gene and the progeny clones that became Neomycin sensitive were confirmed by PCR. The correct disposition of the target vector was further confirmed by sequencing. Clones containing exon 4 flanked by two *loxP* sites and lacking *Neo*^*r*^ were microinjected into C57BL/6 J blastocysts to generate conditional *Ccnd1* exon 4 and 5 knockout mice. Three female mice with germ line transmission were obtained. Two of these mice did not generate any progeny, *i.e*., all floxed mice issued from a single mouse. We sequenced the modified gene and excluded that any epigenetic/genetic event could have modified the *loxP* sites in this founder female mouse.

### Bone marrow reconstitution experiments

CD45.1/45.2 B6 Rag^−/−^ mice were irradiated lethally (1,700 rad) and injected *i.v.* with 5,000 sorted LSKs (2,500 from CD45.1^+^ WT B6 donors and 2,500 from CD45.2 *Vav1-Cre*^*+/−*^*Ccnd1*^*4-5*^*flox*^*+/+*^ mice). Mice were maintained in water supplemented with neomycin one week before and 1 week after irradiation.

### Statistic analysis

Prism software (GraphPad) was used. Two groups were compared with the Student t-test for paired data. *P* values of 0.05 or less were considered significant. Significant differences were *p < 0.05 **p < 0.01 ***p < 0.001.

## Reviewers’ comments

### Reviewer #1 Dr. Manabu Sugai (Kyoto University, Japan)

*We thank the reviewer for the thorough review of our manuscript. To simplify the reading of our answers to the reviewer’s comments, we transcribe directly these comments followed by our answers in italic. The modifications in the Ms. are in red.*

#### Reviewer summary

The manuscript by Chaves-Ferreira et al. addresses the specific function of cyclin D1, which cannot be substituted by other cyclins. The authors found that in addition to full-length cyclin D1, the carboxyl terminal region (exons 4 and 5) of cyclin D1, which the authors designate as the 4–5 proteins, was independently expressed. The 4–5 proteins may promote the transcription of cyclins D2 and D3 as transcriptional activators. The authors noted the variegated phenotypes of cyclin D1d1-3-deficient mice, and after examining the differences between them; they concluded that the expression levels of the 4–5 proteins are the cause of the variegated phenotypes observed in cyclin D1d1-3-deficient haematopoietic cells. The proposed concept is very interesting, but I have few concerns with the methodology and interpretation of the data.

#### Reviewer recommendations to authors

This is a very interesting manuscript that demonstrates the special function of cyclin D1, which is performed by the 4–5 proteins. To understand the physiological significance of the findings, the authors need to show more convincing evidence of the existence of the 4–5 proteins in wild-type cells. The expression of the 4–5 proteins in activated CD8 T cells from cyclin D1d1-3 mice was determined by immunostaining using anti-4-5 D1 mAb (Fig. 4b).

#### Comment 1

To confirm the specificity of this antibody, the expression of the 4–5 proteins in VAV-Cre+/−cyclin D1d4-5:flox/flox CD8 T cells from BM-transferred RAG2−/− mice is required. In addition, same experiment using wild type CD8T cells will be required. It is ambiguous whether the epitope of mAb (DCS6) recognizes exon 3 or the C-terminal part of cyclin D1 (as described in the materials and method section). Therefore, the specificity of DCS6 mAb needs to be determined. The existence of the 4–5 proteins was demonstrated by Western blot analysis of WT-CD8 T cells using DCS6 mAb antibody (Additional file 1: Figure S1). Lysate from VAV-Cre+/−cyclin D1d4-5:flox/flox CD8 T cells is an appropriate negative control to validate the 4–5 proteins that were identified in Additional file 1: Figure S1.

*We think these queries result of an incomplete description of the Abs we used in these studies, what could have lead to misunderstandings. In the revised version of the Ms, we clarified in both the Materials and Methods section (page 19, in red) as well as in the Fig. legends (page 25, Fig.*4*legend, page 29, Additional file**1**: Figure S1 legend, in red) the three different commercial antibodies against cyclin D1 that we used in this study.*

- *The Ab used for WB analysis in Additional file**1**: Figure S1, (to identify the expression of 4–5 by WT CD8 T cells) was****NOT DCS-6****, but a polyclonal rabbit Ab recognizing the C-terminal part of the molecule (from AbCam: ref Ab7958). Although we do not show the results, we obtained similar data with another commercial polyclonal Ab from Cell Signaling (reference 2922) also sold as recognizing the C-terminal part of the D1 molecule.*
- *The mAb DSC-6 (BD pharmingen) that recognizes an epitope in both mouse and human D1 exon 3 was only used for the WB analysis of Vav1-Cre*^*+/−*^*Ccnd1*^*4-5*^*flox*^*+/+*^*mice, shown in Additional file**1**: Figure S6B. This study was performed to show that after 4–5 ablation, the D1 1–3 truncated molecule was yet expressed. This control was important, since it was possible that the 4–5 deletion perturbed the expression of the Ccnd1 gene,* i.e.*, by deleting 4–5 we were just generating a full D1 KO mouse.*
- *For confocal microscopy, we used a rat anti-mouse mAb recognizing the truncated D1 protein coded by exons 4–5, sold by ProMab Biotechnologies, Richmond, USA.*

*All these Abs are commercial, and we expect that these respected companies have tested their specificity. Our WB results confirm the reported specificities, since they recognized the respective proteins at the correct sizes. It is not possible to verify the specificity of all the commercial Abs used for cytometry or WB. Considering the high amount of protein required for sequencing, and the multiple Abs usually used in each Ms. this would be an impossible task.*

*We believe therefore that this query was due to a misunderstanding/poor description of the Abs used and their reported specificity. Besides, the confirmation of the Ab specificity in Vav1-Cre*^*+/−*^*Ccnd1*^*4-5*^*flox*^*+/+*^*cells in BM chimeras is impossible to perform, taking into consideration the minute number of Vav cells recovered from these chimeras, and the absence of CD8 T cells.*

#### Comment 2

The functions of the 4–5 proteins were not directly determined in this manuscript. This is another problem that should be resolved before publication of this manuscript. As shown in Fig. 4a, the expression levels of the 4–5 proteins were well correlated with those of cyclins D2 and D3. However, the authors did not examine whether the expression of cyclins D2 and D3 was due to the expression of the 4–5 proteins. To reveal this, the 4–5 proteins should be introduced into cyclin D1d1-3 LSK cells via lentivirus to estimate the effects in transcription of cyclin D2 and D3 mRNAs. Please show the functional importance of the 4–5 proteins in cyclin D2 and D3 expression.

*We now included in the text (page 12, in red) the direct response to this comment: these experiments cannot provide adequate information. We had already explained the serious limitations of these experiments in detail in Additional file**2**: Note 2- why these experiments cannot give conclusive results; note that we transcribe bellow:*

*Additional file**2**: Note 2: To directly demonstrate that the truncated D1 4–5 molecule has a role in hematopoiesis and in the expression of D2 and D3, two strategies could have been used. One would be to induce the expression of the truncated protein in the hematopoietic precursors from Group I mice and demonstrate that this over-expression would rescue their phenotype, as well as D2 and D3 expression. However, this approach was virtually impossible to perform, and could not give reliable results. It was virtually impossible to perform because Group I mice are quite young (what reduces the total number of cells we can recover from the BM), and virtually devoid of progenitors. Besides, these mice are very rare excluding that we could pool several Group I mice to obtain the enough precursor cells to achieve efficient infection and reconstitution. But more importantly, these experiments could not give straightforward conclusions, because both transduced and non-transduced cells should show compensated phenotypes and normal D2 and D3 expression when studied. Indeed, Injected BM progenitors take one month to generate the various thymocyte sub-populations and two months to reconstitute the peripheral pools. Therefore, injected mice in these experiments should be studied two months after the transfer of progenitors,* i.e. *when these mice will be around 10-weeks-old. However, as we state in the 2*^*nd*^*chapter of results section, Group I mice compensate their Ccnd1 deficiency by 6 weeks of age. Therefore, non-transduced cells should have also have the compensated phenotype when studied in 10-weeks-old mice,* i.e. *would be identical to transduced cells. In transduced cells also showing a compensated phenotype, it would be impossible to attribute this compensation to the expression of Ccnd1 4–5 we had induced or to any other non-identified compensatory mechanism that would be engaged in compensated, non-manipulated mice. Therefore, the only possible strategy to address the role of 4–5 was to generate 4–5 deficient mice. Since this molecule was likely important during embryogenesis, we generated conditional deficient mice.*

*To further detail this point: We agree with reviewer that this kind of experiments could be an important functional clue. However, as explained in the Additional file**2**: Note 2 and described in page 6–7, Ccnd1*^*Δ1–3*^*Group I LSKs have to be recovered from very young mice, which have yet few cells in the BM and are practically devoid of LSK. Cell loss is considerable in sorting rare populations, so we could recover less than 10*^*3*^*LSKs after sorting. This makes transduction and reconstitution very difficult, since Group I mice are rare, so we cannot pool several Group I mice to increase the number of precursors available.*

*But the major problem of this experiment is that readouts would not provide any reliable conclusions. Since Group I mice that survive compensate their deficiencies by rapidly expressing 4–5, it is likely that both transduced and non-transduced cells would express 4–5 and Ccnd2 and Ccnd3 after the long time period required to wait for BM reconstitution. It would be impossible to attribute these expressions to the 4–5 molecule we had introduced or to any other non-identified compensatory mechanism.*

### Reviewer #2 Dr. Wayne Hancock (University of Pennsylvania, USA)

#### Reviewer summary

This is an interesting and well-written report showing that members of the cyclin D family of proteins (D1, D2 and D3) can have distinct, non-redundant roles. This is a fine effort.

#### Reviewer recommendations to authors

No recommendations for additional studies are necessary.

#### Minor issues

None

*We thank reviewer for the thorough review of our manuscript.*

### Answers to re-revisions

Please find enclosed the answers to the reviewer’s comments. To simplify the Editors and Reviewer’s tasks, we transcribe each of the reviewer’s comments, followed by our answer to each comment. The modifications introduced in the text are shown with a yellow background. We thank the reviewer for his work, and hope the modifications introduced meet with his approval.

### Reviewer #1 Dr. Manabu Sugai (Kyoto University, Japan)

#### First comment

Thank you for including the detailed information pertaining to antibodies used in each experiment. I apologize for misunderstanding about the antibodies. However, one of the most important issues to be shown is the convincing evidence of the existence of 4–5 proteins. Commercially available antibodies were tested for specificity by detecting full-length cyclin D1, but almost all the antibodies could also detect the non-specific proteins. As shown in this manuscript, the 4–5 proteins were newly identified, but the bands that formed around 15 kDa using these antibodies were not previously verified by the antibody suppliers. For this reason, the authors need to show that the bands detected by the antibodies (Ab7958, 2992) genuinely represent the 4–5 proteins. To support this, mouse embryonic fibroblasts (MEF) derived from cyclin D1d4-5:flox/flox mice would be useful. Cre-transduced cyclin D1d4-5:flox/flox MEF are an appropriate negative control for verifying the 4–5 proteins. The authors need to verify the existence of the 4–5 proteins by immunoblotting assay using three antibodies (Ab7958 from Abcam, 2992 from Cell Signaling Technology, and rat anti-mouse mAb from ProMab Biotechnologies) and compare the lysates from Cre-transduced and non-transduced cyclin D1d4-5:flox/flox MEF. Confocal microscopic images of Cre-transduced and non-transduced cyclin D1d4-5:flox/flox MEF are also required.

1. To summarise comment #1: The reviewer objects that we did not demonstrate that wild type cells and the cells from Ccnd11-3 compensated mice expressed the protein coded by 4–5 exons of D1 because we did not demonstrate the specificity of the Abs using an appropriate negative control. 2) To summarise this comment, the reviewer indicated that the truncated D1 molecule (cyclin D11-3), which is produced by an ablation of 4–5, might lose some functions of the full length cyclin D1.

We believe our Fig. 4 demonstrates clearly that “compensated” mice express a truncated D1 4–5 molecule. In this experiment we used a mAb raised against 4–5 to study 4–5 expression. The commercial firm that sold us this Ab, send us data showing that this Ab does not cross-react with D2 and D3. It was however possible that the Ab would recognize any other protein expressed by the cell, what would invalidate our results.

Our controls for specificity were the cells from Group I, fully D1 deficient mouse. If this mAb would recognize other proteins besides D1, one would expect some labeling in cells from Group I mice, but no labeling was detected, demonstrating that this mAb only recognizes D1. By contrast, this mAb labels all cells of compensated mice. Since compensated mice are D1^1–3^ deficient, this mAb cannot label 1–3 that is not there, it must label 4–5. We conclude that compensated mice do express 4–5 proteins, which migrate to the nucleus after T cell activation and bind to the DNA. **We now add this comment to the revised Ms, page 9 last paragraph and page 10 first paragraph**.

Since in contrast to this mAb, the polyclonal Abs used in WB recognize multiple cellular proteins, we tuned down our conclusions concerning the expression of 4–5 in WT cells. We now state that the WB suggests the independent expression of 4–5 in WT cells (see page 10, second paragraph).

The experiments suggested by the reviewer would require the back-cross of the Vav-Cre^+^ 4–5 ^flox/flox^ mice to WT mice to eliminate Vav Cre, followed by the intercross of 4–5 ^flox+/−^ mice to obtain ^flox/flox^ mice, the preparation of embryonic fibroblast lines, the transfection of these lines with Cre. These experiments required a vast breeding program that would take months and could be compromised by the serious breeding problems of the Vav-Cre^+^ 4–5 ^flox/flox^ line. We here show that they are not required to demonstrate the important point that compensated mice express the 4–5 protein.

#### Second comment

The authors mentioned that the 4–5 proteins may promote the transcription of cyclins D2 and D3 and rescue the defect observed in cyclin D1d1–3-deficient hematopoietic cells. Although the idea is of great significance, the authors fail to describe whether the expression of the 4–5 proteins is a cause or a consequence in rescuing this defect. As shown in Fig. 4a, the expression levels of the 4–5 proteins were well correlated with those of cyclins D2 and D3. According to these data, the authors can also conclude that the expression levels of cyclins D2 and D3 are the cause of the rescued phenotypes observed in cyclin D1d1–3-deficient hematopoietic cells. Therefore, the authors need to demonstrate that the enforced expression of the 4–5 proteins rescue cyclin D1d1–3-deficient hematopoietic cells. These data are essential to get the manuscript published.

2) It appears this conclusion very unfair to this extensive work. We have extensive data demonstrating directly the role of D1 in hematopoiesis including the block in hematopoiesis by eliminating D1 by *RNAi*. The review cannot ignore all this data, and does not context it. The only point he objects is the role of 4–5. We had previously explained why the enforced expression of 4–5 in D1 deficient mice was impossible to perform and could not lead to conclusive results in an additional note. We now also include a summary of these points in the text, page 12, last paragraph.

#### Second comment bis

In this regard, **the generation of transgenic mice that express the 4–5 proteins in hematopoietic cells is required.**

- **Rather than generating a transgenic mouse, we enforced the expression of 4–5 in WT LSKs, what should give equivalent results, but was faster to perform.** These cells were co-transferred together with Mock transduced cells into lethally irradiated Rag- mice. We compared cell division by BrdU incorporation in both populations of EFGP^+^ transduced cells. We show in the new Table 1 (page 45), that enforced expression of 4–5 significantly increases division rates (see also page 12, last paragraph, page 13, first paragraph), thus correlating directly the over-expression of 4–5 to cell division (see page 13, first paragraph).

#### Third comment

The authors examined the phenotype of VAV-Cre^+^/cyclin D1d4-5^flox/flox^ mice to study the function of the 4–5 proteins. This approach is not suitable to evaluate the 4–5 proteins, because cyclin D1 function might be affected by the deletion of exon 4 and 5. Therefore, they cannot exclude the possible involvement of full-length cyclin D1 in the phenotypes observed in these mice.

3) To summarize this comment, the reviewer objects that our experiments of BM competition, (where BM cells from Vav-Cre^+^ 4–5 ^flox/flox^ mice behaved as full D1 deficient cells) are not valid, since the conditional ablation of 4–5 could have perturbed the expression of the full D1 molecule. In this perspective, cells from Vav-Cre^+^ 4–5 ^flox/flox^ mice, rather than expressing a truncated D1 molecule coded by exons 1–3 could be fully D1 deficient.

We had demonstrated clearly that in creating the Vav-Cre *Ccnd1*^*4-5*^*flox*^*+/+*^ mice we were not just generating a fully D1 deficient mouse: a) the experimental strategy we used to generate these mice was designed to allow the stable expression of 1–3 (see page 13, second paragraph, in yellow); b) We clearly demonstrated that these cells were not just full D1 deficient in Additional file 1: Figure S6B. The western blots shown in this Fig. clearly show the presence of a 1–3 band, labeled with mAb DCS-6 (see page 13, last paragraph and page 14 first paragraph).

The reviewer could object that we do not know the specificity of this Ab, and that the commercial firm that sells it did not tested it. Two firms sell this Ab, B&D and Cell Signaling. While B&D provides no information, Cellsignaling (reference #2926) describes the polypeptide located in exon 3 used for immunization, and the absence of cross-reactivity with D2 and D3. **IMPORTANTLY** I have a long-term experience with this mAb. Prof. Sherr advised me to use it, due to its high quality. We used it extensively in one previous publication that we now quote (Veiga-fernandes & B. Rocha, Nat Immunol 2004). We demonstrated no cross-reactivity with D2 and D3. Importantly, this mAb **DOES NOT CROSS-REACT WITH ANY OTHER CELL PROTEIN**. The WB analysis of total cell proteins only shows the D1 band. Therefore, our results demonstrate that the Floxed mice express the truncated D1 molecule corresponding to exons 1–3.

### Reviewer #2 Dr. Wayne Hancock (University of Pennsylvania, USA)

#### Reviewer summary

This is an interesting and well-written report showing that members of the cyclin D family of proteins (D1, D2 and D3) can have distinct, non-redundant roles. This is a fine effort.

#### Reviewer recommendations to authors

No recommendations for additional studies are necessary.

#### Minor issues

None

*We thank both reviewers for the thorough review of our manuscript.*

## Additional files

Additional file 1: Figure S1. Expression of C-terminal D1 polypeptides in WT mice. **Figure S2:** Expression of activated-caspase-3 in hematopoietic lineage cells from *Ccnd1* ^Δ1–3^ mice. **Figure S3:** Expression of Bcl-2 in hematopoietic lineage cells from *Ccnd1* ^Δ1–3^ mice. **Figure S4:** Efficiency and specificity of D1 RNA interference. **Figure S5:** The Targeting vector used to generate *Vav1-Cre* ^*+/−*^ *Ccnd1* ^*4-5*^ *flox* ^*+/+*^ mice. **Figure S6:** Characterization of *Vav1-Cre* ^*+/−*^ *Ccnd1* ^*4-5*^ *flox* ^*+/+*^ mice. (PDF 1744 kb) Additional file 2: Note 1: No alterations in cell death in Ccnd1D1‑3 mice. Note 2: Strategies that could be used to demonstrate that the truncated D1 4–5 molecule has a role in hematopoiesis and in the expression of D2 and D3. Note 3: The breeding of *Vav1-Cre* ^*+/−*^ *Ccnd1* ^*4-5*^ *flox* ^*+/+*^ mice generated even less viable progeny than *Ccnd1* ^Δ1–3^ breeding. (DOC 25 kb) Additional file 3: Chaves-Ferreira et al, Additional Materials and Methods. (DOC 31 kb)

## Abbreviations

BM: bone marrow
BrdU: Bromodeoxyuridine (5-bromo-2′-deoxyuridine)
*Ccnd1*^Δ1-3^: deletion of exons 1–3 in cyclin D1 gene (*Ccnd1*)
CDK: cyclin dependent kinases
CLP: common lymphocyte progenitors (Lineage^−^IL-7R^+^Sca-1^+^CD117^+^)
EGFP: enhanced green fluorescent protein reporter
ETP: CD44^+^ CD25^−^ TN1 early thymocyte progenitors, subdivided by their expression of CD24 and CD117
LSK: hematopoietic stem cells (Lineage^−^ Sca-1^+^CD117^+^)
mAb: monoclonal antibody
OP-9: murine bone marrow stroma cell line
PFA: Paraformaldehyde
Rb: Retinoblastoma protein
RNA*i*: RNA interference
SDS-PAGE: sodium dodecyl sulfate polyacrylamide gel electrophoresis
TN: triple negative (CD4^−^CD8^−^CD3^−^ Lineage^−^) thymocytes
WT: wild type mice
