# Supplementary material for: The cyclin D1 carboxyl regulatory domain controls the division and differentiation of hematopoietic cells
Source: Biol Direct. 2016 Apr 29;11:21. doi: 10.1186/s13062-016-0122-9 (PMC4851827; doi:10.1186/s13062-016-0122-9)
Supplement: Additional file 1: Figure S1. — Expression of C-terminal D1 polypeptides in WT mice. Figure S2: Expression of activated-caspase-3 in hematopoietic lineage cells from Ccnd1 Δ1–3 mice. Figure S3: Expression of Bcl-2 in hematopoietic lineage cells from Ccnd1 Δ1–3 mice. Figure S4: Efficiency and specificity of D1 RNA interference. Figure S5: The Targeting vector used to generate Vav1-Cre +/− Ccnd1 4-5 flox +/+ mice. Figure S6: Characterization of Vav1-Cre +/− Ccnd1 4-5 flox +/+ mice. (PDF 1744 kb) [file 13062_2016_122_MOESM1_ESM.pdf]

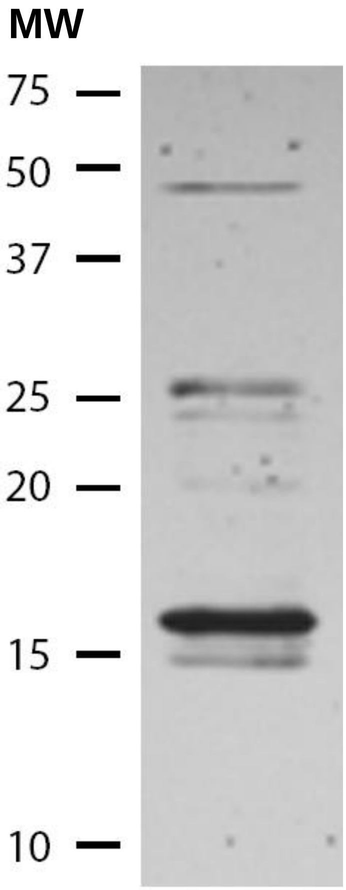

Chaves-Ferreira *et al*, **Additional Figure 1**

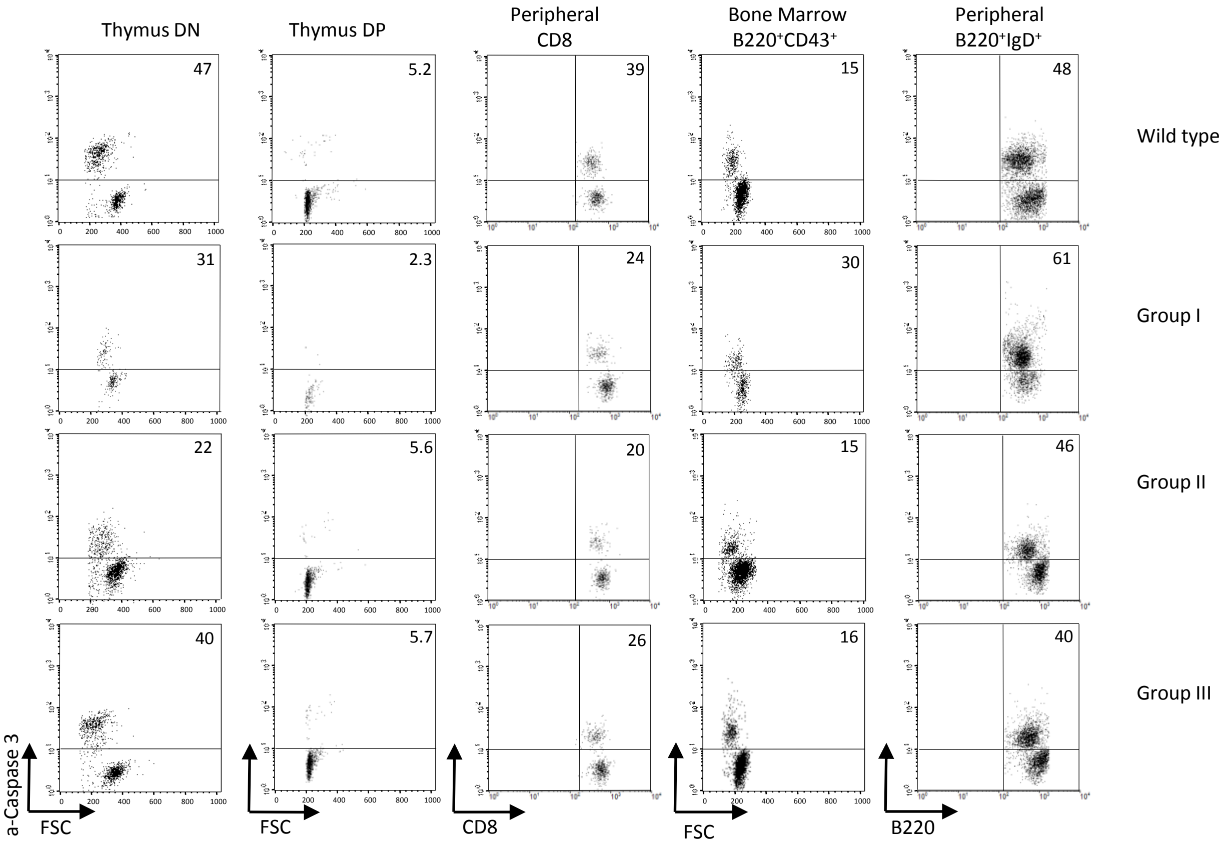

Chaves-Ferreira *et al*, **Additional Figure 2**

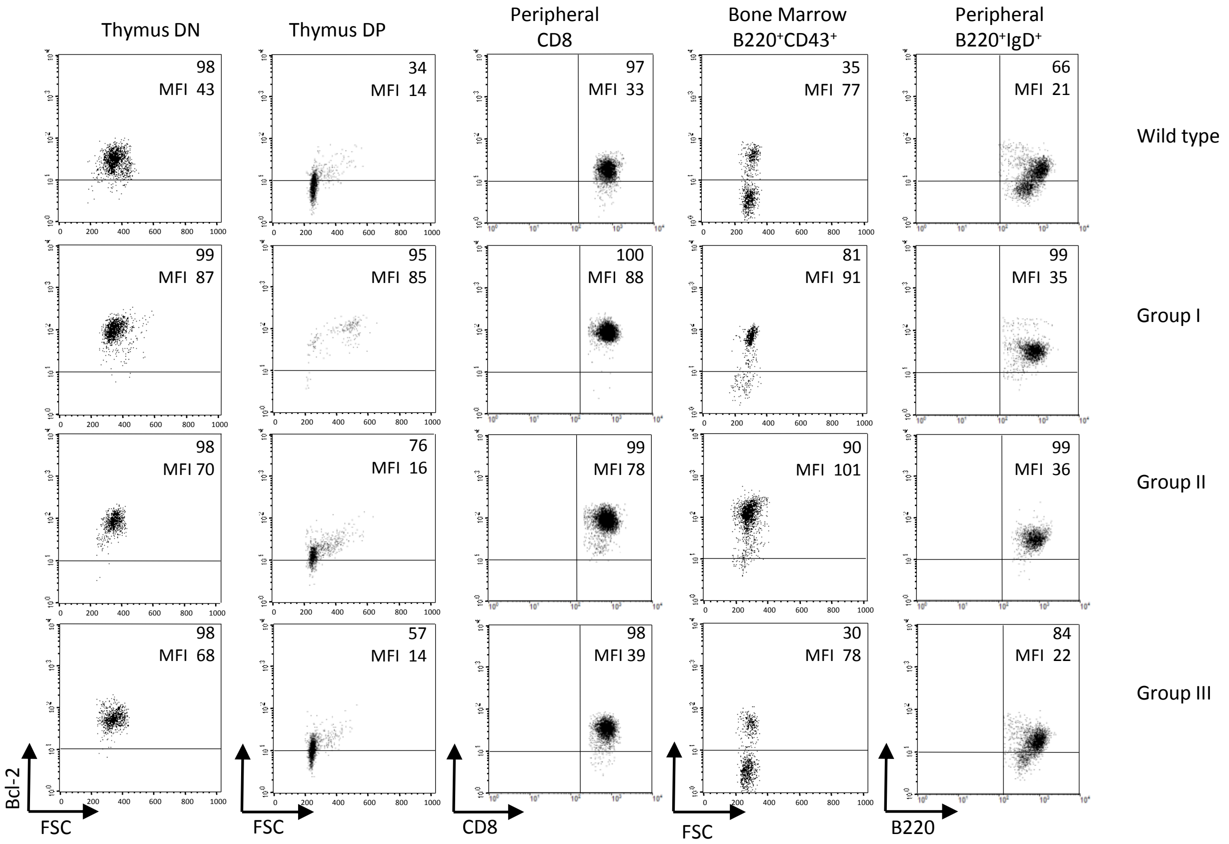

Chaves-Ferreira *et al*, **Additional Figure 3**

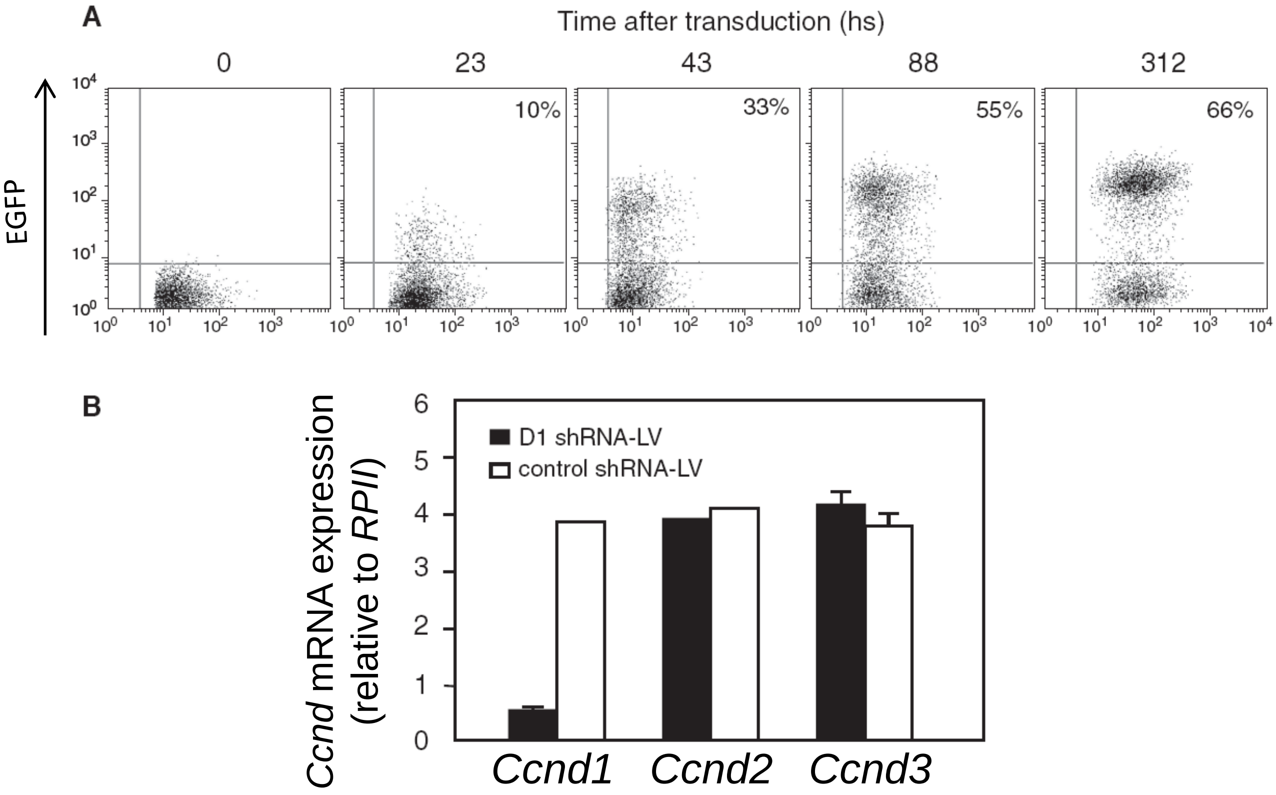

Chaves-Ferreira *et al*, **Additional Figure 4**

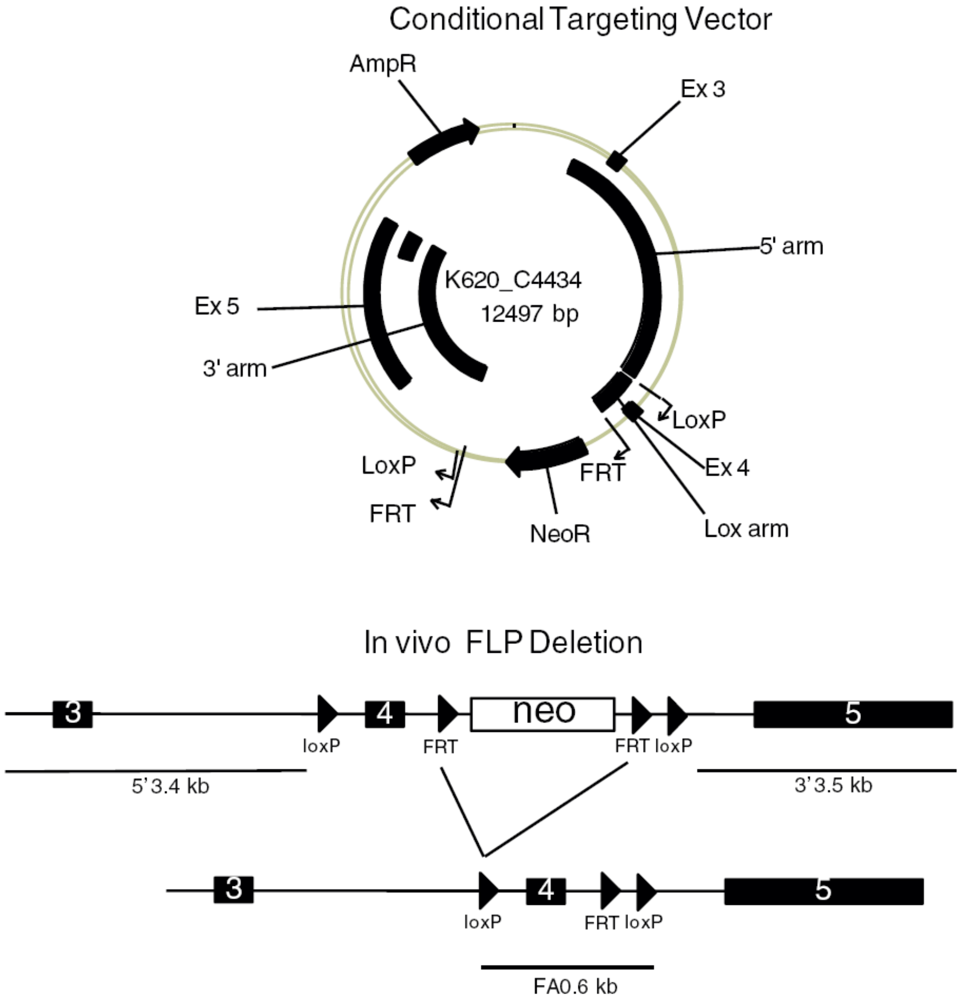

Legend:
5': 5' homology arm; FA: floxed fragment; 3': 3' homology arm
This schematic representation is not on scale

Chaves-Ferreira *et al*, **Additional Figure 5**

A

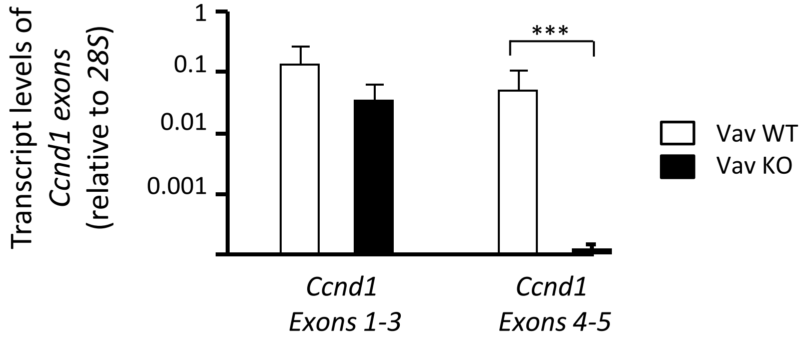

B

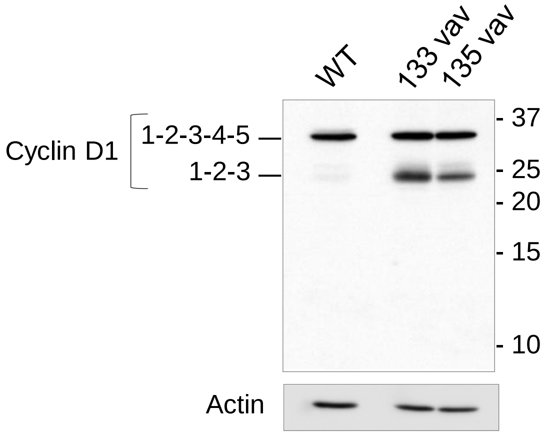

C

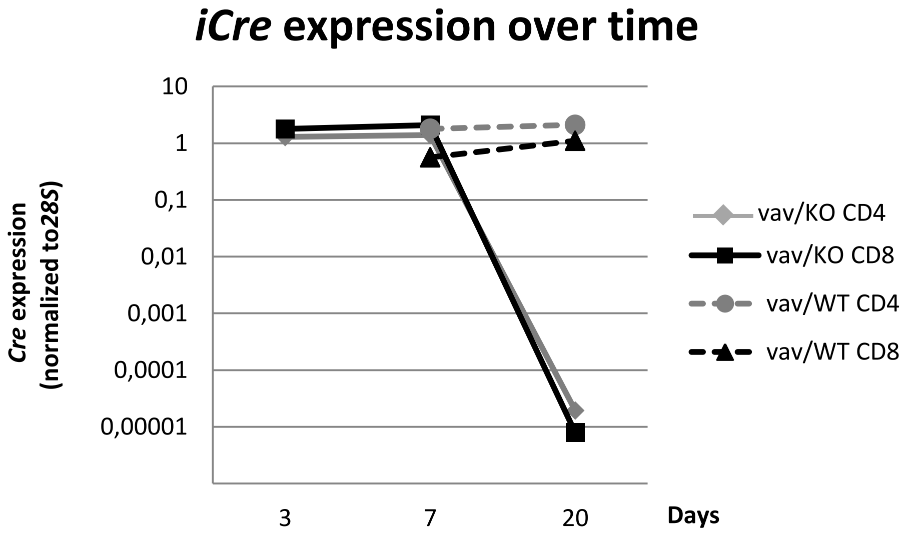

Chaves-Ferreira *et al*, **Additional Figure 6**
