# Supplementary material for: The cyclin D1 carboxyl regulatory domain controls the division and differentiation of hematopoietic cells
Source: Biol Direct. 2016 Apr 29;11:21. doi: 10.1186/s13062-016-0122-9 (PMC4851827; doi:10.1186/s13062-016-0122-9)
Supplement: Additional file 2: — Note 1: No alterations in cell death in Ccnd1D1‑3 mice. Note 2: Strategies that could be used to demonstrate that the truncated D1 4–5 molecule has a role in hematopoiesis and in the expression of D2 and D3. Note 3: The breeding of Vav1-Cre +/− Ccnd1 4-5 flox +/+ mice generated even less viable progeny than Ccnd1 Δ1–3 breeding. (DOC 25 kb) [file 13062_2016_122_MOESM2_ESM.doc]

**Additional Note 1**: An increased cell death could also contribute to the reduction of precursor frequencies in *Ccnd1*^Δ1‑3^ mice. When compared to WT cells, *Ccnd1*^Δ1‑3^ T lineage cells showed lower levels of activated caspase-3 labeling, and higher levels of Bcl-2 expression, actually indicating reduced death rates **(Additional Fig. 2 & 3)**. In Group I mice, B lineage cells should also survive since although they had higher frequency of activated caspase-3^+^ cells, their Bcl-2 expression was up-regulated. In other Groups, these parameters were identical to WT cells. Altogether, these results indicate that the main cause of the failure of hematopoietic precursors to differentiate in *Ccnd1*^Δ1‑3^ Group I mice is their inability to divide.

**Additional Note 2*:*** To directly demonstrate that the truncated D1 4-5 molecule has a role in hematopoiesis and in the expression of D2 and D3, two strategies could have been used. One could be to induce the expression of the truncated protein in the hematopoietic precursors from Group I mice, and demonstrate that this expression would rescue their phenotype. However, this approach was virtually impossible to perform, and could not give reliable results. It was virtually impossible to perform because Group I mice are quite young (what reduces the total number of cells we can recover from the BM), and virtually devoid of progenitors. Besides, these mice are very rare excluding that we could pool several Group I mice to obtain the enough precursor cells to achieve efficient infection and reconstitution. But more importantly, these experiments were unlikely to give straightforward conclusions, because both transduced and non-transduced cells should show compensated phenotypes when tested. Indeed, these mice had to be studied several weeks after LSK injection. However, as we state in the 2^nd^ chapter of results section, Group I mice compensate their *Ccnd1* deficiency by 6 weeks of age. Therefore, non-transduced cells should have the compensated phenotype when studied *i.e.* would be identical to transduced cells. Moreover, if transduced cells showed a compensated phenotype, it would be impossible to discriminate if this compensation was due to the expression of Ccnd1 4-5 we had induced or to any other compensatory mechanism that would be engaged in compensated, non-manipulated mice. Therefore, the only possible strategy to address the role of 4-5 was to generate 4-5 deficient mice. Since this molecule was likely important during embryogenesis, we generated conditional deficient mice.

**Additional Note 3**: The breeding of *Vav1-Cre^+/-^Ccnd1^4-5^flox^+/+^* mice generated even less viable progeny than *Ccnd1*^Δ1-3^ breeding. One of the possibilities explaining this increased prenatal death could be due to the very reduced capacity of *Vav1-Cre^+/-^Ccnd1^4-5^flox^+/+^* mice to compensate for the absence of *Ccnd1 4-5*, when compared to *Ccnd1*^Δ1-3^ mice. Indeed, as we show in Additional Fig. 6, *Vav1-Cre^+/-^Ccnd1^4-5^flox^+/+^* mice compensate the *Ccnd1 4-5* deficiency by selecting cells that fail to express *Cre*, and therefore by expressing the full length WT D1 protein. However, precursors failing to express *Cre* must be very rare since in non-selecting conditions (when *Vav* mice are crossed with *Rosa-flox-STOP-flox GFP* mice) they are not detected-all hematopoietic lineage cells express GFP [22]. By contrast, the selection of precursors expressing *Ccnd1 4-5* in *Ccnd1*^Δ1-3^ mice must be relatively more efficient, since WT cells also express these truncated proteins.
