# Supplementary material for: The cyclin D1 carboxyl regulatory domain controls the division and differentiation of hematopoietic cells
Source: Biol Direct. 2016 Apr 29;11:21. doi: 10.1186/s13062-016-0122-9 (PMC4851827; doi:10.1186/s13062-016-0122-9)
Supplement: Additional file 3: — Chaves-Ferreira et al, Additional Materials and Methods. (DOC 31 kb) [file 13062_2016_122_MOESM3_ESM.doc]

**Chaves-Ferreira et al, Additional Materials and Methods**

**Monoclonal antibody against the 4-5 D1 polypeptide**

The mAb against 4-5 D1 was produced by ProMab Biotechnologies (Richmond, USA). Briefly, rats were immunized with the synthetic peptide

MHHHHHHVKFISNPPSMVAAGSVVAAMQGLNLGSPNNFLSCYRTTHFLSRVIKCDPDCLRACQEQIEALLESSLRQAQQNVDPKATEEEGEVEEEAGLACTPTDVRDVD, present in the D1 4-5 domain and not in other proteins, including D2 and D3. Ig^+^ Hybridomas were screened by ELISA for the reactivity against this peptide and the absence of reactivity against D2 and D3.

**RT-qPCR and Primers**

The sequences (5’ to 3’) of primers used for qPCR were the following:

*muRNA polymerase II*: forward: catctcagaatgctagcacca; reverse: tgaatccaaaggtcgtttctg

*muCcdn1*: forward: tgctgcaaatggaactgctt ; reverse: ccacaaaggtctgtgcatgct

*muCcdn2*: forward: caccgacaactctgtgaagc; reverse: atgaagtcgtgaggggtgac

*muCcdn3*: forward: ctacttccagtgcgtgcaaa; reverse: agccagagggaagacatcct

*muCcdn1(exon4-5):* forward: cccaacaacttcctctcctg; reverse tcagatgtccacatctcgca

*GAPDH-* forward: cctcgtcccgtagacaaaatg ; reverse: tgaaggggtcgttgatggc

Quantitative RT-PCR was performed as previously described (Peixoto et al. 2004). Expression levels for all genes are expressed in arbitrary units relative to the housekeeping gene. Standard deviations were calculated from triplicates.

**Primers used for Cloning of *Ccnd1* exons 4-5**

Forward Cloning Primer: actcaattggccatggtgaagttcatttccaaccc

Reverse Cloning Primer : tgcgagatgtggacatctgagcctacgtatct

**shRNA**

Oligonucleotides were purchased from Invitrogen Life Technologies (Cergy Pontoise, France). The forward sequences of small hairpins were the following:

D1 (646) 5’ cgcgtcccccctgggcagccccaacaacttcaagagagttgttggggctgcccaggtttttggaaat 3’

Scrambled Control 5’cgcgtccccacgggccgcccttaatacattcaagagatgtattaagggcggcccgttttttggaaat 3’

Forward and reverse oligonucleotides were annealed in 500 mM potassium acetate, 150 mM Hepes pH 7.4, 10 mM magnesium acetate. shRNA sequences were designed in a first instance by using the ambion free-software (no longer available) and selected according to the rules reported to predict efficient sequences.
